# Ultra-small bacteria and archaea exhibit genetic flexibility towards groundwater oxygen content, and adaptations for attached or planktonic lifestyles

**DOI:** 10.1038/s43705-023-00223-x

**Published:** 2023-02-17

**Authors:** Emilie Gios, Olivia E. Mosley, Louise Weaver, Murray Close, Chris Daughney, Kim M. Handley

**Affiliations:** 1 School of Biological Sciences, The University of Auckland, Auckland, New Zealand; 2 Institute of Environmental Science and Research, Christchurch, New Zealand; 3 GNS Science, Lower Hutt, New Zealand; 4 Present Address: NINA, Norwegian Institute for Nature Research, Trondheim, Norway; 5 Present Address: NatureMetrics Ltd, Surrey Research Park, Guildford, UK; 6 Present Address: NIWA, National Institute of Water and Atmospheric Research, Wellington, New Zealand

**Keywords:** Water microbiology, Microbial ecology

## Abstract

Aquifers are populated by highly diverse microbial communities, including unusually small bacteria and archaea. The recently described Patescibacteria (or Candidate Phyla Radiation) and DPANN radiation are characterized by ultra-small cell and genomes sizes, resulting in limited metabolic capacities and probable dependency on other organisms to survive. We applied a multi-omics approach to characterize the ultra-small microbial communities over a wide range of aquifer groundwater chemistries. Results expand the known global range of these unusual organisms, demonstrate the wide geographical range of over 11,000 subsurface-adapted Patescibacteria, Dependentiae and DPANN archaea, and indicate that prokaryotes with ultra-small genomes and minimalistic metabolism are a characteristic feature of the terrestrial subsurface. Community composition and metabolic activities were largely shaped by water oxygen content, while highly site-specific relative abundance profiles were driven by a combination of groundwater physicochemistries (pH, nitrate-N, dissolved organic carbon). We provide insights into the activity of ultra-small prokaryotes with evidence that they are major contributors to groundwater community transcriptional activity. Ultra-small prokaryotes exhibited genetic flexibility with respect to groundwater oxygen content, and transcriptionally distinct responses, including proportionally greater transcription invested into amino acid and lipid metabolism and signal transduction in oxic groundwater, along with differences in taxa transcriptionally active. Those associated with sediments differed from planktonic counterparts in species composition and transcriptional activity, and exhibited metabolic adaptations reflecting a surface-associated lifestyle. Finally, results showed that groups of phylogenetically diverse ultra-small organisms co-occurred strongly across sites, indicating shared preferences for groundwater conditions.

## Introduction

Aquifers are biologically complex environments, encompassing highly diverse microbial communities [1]. Microbial life in the subsurface is estimated to constitute 15% of the Earth’s total biomass [2], but remains mostly uncultivated and largely unexplored. Recent metagenomic investigations led to the identification of the bacterial Candidate Phyla Radiation (CPR) [3] along with the archaeal DPANN radiation (initially proposed to include Diapherotrites, Parvarchaeota, Aenigmarchaeota, Nanoarchaeota, and Nanohaloarchaeota, and expanded to include candidate phyla such as Micrarchaeota and Woesearchaeota) [4, 5]. The CPR encompasses over 70 candidate phyla [1, 6], including the Patescibacteria superphylum (comprising Microgenomates, Parcubacteria and Gracilibacteria) proposed by Rinke et al. [4]. The monophyletic CPR group were subsequently proposed to be subsumed into a single phylum, Patescibacteria, following taxonomic rank normalization to relative evolutionary divergence as part of the Genome Taxonomy Database (GTDB) project [7]. While the term CPR is more commonly used [8–10], both terms (CPR and Patescibacteria) interchangeably define a major bacterial group whose discovery dramatically expanded our view of the microbial phylogenetic tree [3]. Patescibacteria and DPANN have been detected in diverse environments, including hot springs [11–13], animal microbiomes [14–16], and permafrost [17], in addition to aquifers, where they can account for a significant fraction of microbial diversity and abundance [1, 18, 19].

Patescibacteria and DPANN are characterized by ultra-small cells (<0.4 μm in diameter) [19–22] and genomes (~1 Mbp), likely due to genome streamlining [23]. Environment-recovered genomes of ultra-small prokaryotes exhibit a reduced metabolism with limited biosynthetic capacities, including the inability to synthesize most amino acids or lipids [9]. Nonetheless, some studies suggest that these organisms play key roles in subsurface carbon and nitrogen cycling [18, 24]. Genomically-inferred auxotrophies indicate a reliance on other organisms for basic cellular functions, as shown for *Nanoarchaeum equitans* (Nanoarchaeota) [25] and *Candidatus* Saccharibacteria (previously TM7) [15]. The vast majority of these auxotrophies, and involvement in symbiotic or parasitic relationships, are predictions [6, 9]. However, the epibiotic nature of some ultra-small prokaryotes has been illustrated by microscopy [10, 12, 25]. Recent research has also demonstrated correlations in the abundance of ultra-small organisms and known [25] or predicted [10] hosts in the environment.

While most of the diversity of ultra-small prokaryotes has been described from aquifers [1, 10, 21], the global distribution, ecological niches, function, and biological interactions of ultra-small microorganisms in groundwater remain poorly characterized. To date, information about Patescibacteria derives almost exclusively from metagenomic and amplicon analyses from a small number of aquifer locations (mostly in the USA and more recently also Germany) [6, 18, 26]. Although a recent metatranscriptomic study showed that many candidate phyla were active community members in the deep subsurface [27], proteomic and transcriptomic studies, providing insights into their activity, are chiefly lacking due to the difficulty in obtaining sufficient biomass from naturally oligotrophic groundwater. Patescibacteria and DPANN are also mostly described from anoxic or low-oxygen habitats, and characterised by anaerobic metabolisms [6, 9]. They are thought to rely solely or heavily on fermentation. However, recent research also indicates that Patescibacteria can be oxygen tolerant, enabling them to colonise oxic habitats, such as soils [28]. One subsurface parcubacterium even harbours a complete electron transport chain, suggesting it is capable of aerobic respiration [29], and a small number of DPANN have near-complete electron transport chains [9]. This suggests the possibility that these organisms occupy a broad range of redox conditions in aquifers.

Here we tested the prediction that Patescibacteria and DPANN are common to aquifer systems, and transcriptionally active across distinct redox conditions. Given compositional differences reported between planktonic and biofilm fractions of other groundwater prokaryotes [30], we also determined the compositional, genetic and transcriptional differences between planktonic and sediment-associated ultra-small community fractions. To assess the distribution, metabolic activity and environmental preferences of aquifer microbiomes, we collected 81 groundwater samples from 10 different aquifers spread over hundreds of kilometers [30, 31]. This enabled us to determine associations between the ultra-small fraction and groundwater physicochemistry. Sixteen metagenomes and six metatranscriptomes, generated from groundwater and sediment-enriched groundwater, were analyzed to identify genomic and metabolic traits and transcriptional activities associated with oxic and dysoxic conditions, and to compare planktonic and biomass-rich surface-attached communities. We present insights into ultra-small prokaryote gene expression in natural and nutrient-enriched groundwater, showing that they are active, but transcriptionally distinct, members of aquifer microbial communities. Results contribute to further define environmental preferences of Patescibacteria, Dependentiae and DPANN genomes, and show that aquifers across distant parts of the globe are colonized by these unusual organisms.

## Materials and methods

### Sample collection

Eighty-one groundwater samples were collected from 59 wells across four New Zealand regions (Auckland, Waikato, Wellington and Canterbury) from sand/gravel (×71), ignimbrite (×6), and one each of basalt, gravel/peat, sand/silt and shell-bed aquifers (Table S1). Wells were purged (3–5 times bore volumes). Then 3–90 L of groundwater was filtered per sample. Biomass was captured on 0.22 μm mixed cellulose ester (MCE) filters, after passing through a 1.2 μm MCE pre-filter, using a 142 mm filter holder (Merck Millipore Ltd., Cork, Ireland). Samples were preserved in RNAlater (ThermoFisher Scientific, Waltham, MA, USA), transported on dry ice, and stored at −80 °C.

Eight Canterbury wells were sampled for meta-omics. After collecting a groundwater sample (as above), a second sample per well was collected following low frequency sonication (2.43 kW for 2 min) to induce biofilm (and sediment) detachment from the surrounding aquifer, using a custom sonication device as described previously [32]. 0.5–15 L of sediment (and hence biomass) enriched groundwater was filtered as above.

For size fraction analysis, groundwater (20 L) and sediment-enriched groundwater (1 L) from well E1 were serially filtered through 1.2, 0.22 and 0.1 μm MCE filters as above (Table S1). In parallel, groundwater (2 L) was concentrated to ~10 mL and sediment-enriched groundwater (900 mL) to ~30 mL using tangential flow filtration (Vivaflow 200 with 5000 molecular weight cutoff cassettes, ~1.5 nm pore size) (Sartorius, Goettingen, Germany). Samples were flash frozen using liquid nitrogen, and stored at −80 °C.

### Groundwater geochemistry and heterotrophic plate counts

Groundwater physicochemical parameters (Table S1) were measured as described by Mosley et al. [30] (also see details on methods in Supplementary Materials). Groundwater and sediment-enriched groundwater from the Canterbury meta-omics series were analyzed for heterotrophic cell counts using pour-plating assays. Samples, or dilutions thereof were pipetted (in triplicate) onto sterile Petri dishes and 15–20 mL 10% R2A agar (LAB203, Fort Richards, New Zealand) were added. Plates were swirled to mix samples into the agar and allowed to set before being incubated, inverted, at 21 ± 1 °C for 6 ± 1 days. All colonies on plates were enumerated by eye and recorded as colony forming units (CFU) per mL for each sample.

### Nucleic acid extraction, sequencing, and processing

Genomic DNA was extracted from all 81 groundwater samples for 16S rRNA gene amplicon generation (using 515F [33] and 806R [34] primers), and a subset of 16 from Canterbury were used for metagenomics (gwj01-gwj16). Total RNA was extracted from six Canterbury samples for transcriptomics (gwj09, gwj11, gwj13–16). Methods for nucleic acid extractions, PCR, sequencing (amplicon, metagenome, metatranscriptomes), read trimming, and metagenome-assembled genome (MAG) generation and curation are as described by Mosley et al. [29] (also see details in Supplementary Materials).

MAGs sharing >99% average nucleotide identity (ANI) were dereplicated across all assemblies using dRep v2.0.5 [35]. Completeness and contamination of ultra-small prokaryote genomes was assessed based on the presence of 51 bacterial single-copy genes (SCGs) for Dependentiae, 43 SCGs for Patescibacteria and 38 archaeal SCGs for DPANN [1]. For other prokaryotes, completeness was estimated using CheckM v1.0.13 [36]. To determine coverage, reads were mapped onto the de­replicated MAGs using Bowtie v1.2.0 [37] (Supplementary Materials). Sample-specific genome relative abundance was calculated by normalizing based on the highest read count between samples [22]. Quality trimmed transcriptome reads were mapped to dereplicated MAGs as described in Supplementary Materials. Read counts were normalized via a modified transcripts per kilobase per million reads mapped (modified-TPM) formula using library size: (number of reads mapped to gene)*(1000/gene length)*(1000000/library size).

### 16S rRNA gene amplification, sequencing and processing

PCR amplification of 16S rRNA genes with 515F [33] and 806R [34] primers and sequencing (2 × 250 bp, Illumina MiSeq V2 platform, San Diego, CA, USA) was undertaken as described previously [38], and OTUs and ASVs were generated (details in Supplementary Materials).

### RNA extraction, sequencing and metatranscriptome analysis

Total RNA was extracted from six Canterbury samples (gwj09, gwj11, gwj13–16), from which metatranscriptomes were generated and analyzed as described previously [30] (details in Supplementary Materials).

### Metabolic predictions

For metabolic predictions, only genomes with completeness >70% and contamination <5% were considered. Protein-coding gene sequences were predicted using Prodigal v2.6.3 [39] (alternative genetic code 25 for Gracilibacteria [40]). Genes were annotated using USEARCH [41] against UniRef100 [42], Uniprot [43] and KEGG [44] databases (*e*-value ≤ 0.001 and identity ≥50%), and HMMs against PFAM [45] and TIGRFAM [46] databases (*e*-value ≤ 0.001). Annotation with eggNOG-mapper v2 [47] and the eggNOG v5.0 database [48] used default parameters. Metabolic pathways were reconstructed using KEGGDecoder v1.1 [49] from annotations generated with KofamKOALA (*e*-value ≤ 0.001). Signal peptides were predicted using SignalP v5.0 [50], with the following options based on previous membrane properties predictions and direct observations: -org arch for DPANN archaea, -org gram- for Dependentiae [51] and -org gram+ for Patescibacteria [8, 16]. Principal Component Analysis (PCA), based on Clusters of Orthologous Genes (COG) metabolic categories compositions (from eggNOG-mapper), was performed using the ggfortify v0.4.11 [52] and ggConvexHull v0.1.0 [53] R packages.

### Genome taxonomy

Taxonomic classification of MAGs was performed using the Genome Taxonomy Database Toolkit (GTDB-Tk, v0.2.1) [54] with database release 89. Maximum likelihood trees were constructed using 120 bacterial and 122 archaeal concatenated marker genes with IQ-TREE v1.6.12 [55] using 100 bootstraps, and ModelFinder [56] best-fit model LG + F + G4 for bacteria and LG + F + I + G4 for archaea. Trees were visualized and annotated with iTOL [57]. MAGs (≥80% complete) were compared to GTDB representative genomes (downloaded 20-Jan-2020) by calculating average amino-acid identities (AAI) for blastp matches sharing ≥30% identity over ≥70% of alignment length.

### Microbial community analysis

Community distributions in association with chemical parameters were visualized using the ggtern R package v3.3.0 [58]. R package ggplot2 v3.3.3 [59] was used for plotting unless specified. Ordinations illustrating Bray–Curtis dissimilarities based on 16S rRNA gene amplicon relative abundance and genome coverage were performed using distance-based redundancy analysis (dbRDA) with the vegan package v2.5–6 [60]. Significant environmental factors, explaining beta-diversity, were determined by a permutation test (*p* < 0.05, permutations = 999) and plotted onto the dbRDA. To determine the co-occurrence of ultra-small prokaryote MAGs across sites, a network was built using the igraph R package v1.1.2 [61]. EdgeR package v3.32.1 [62] was used to calculate log fold changes in gene expression between groundwater conditions based on unnormalised transcript counts.

## Results and discussion

### Ultra-small prokaryotes were prevalent across diverse aquifer lithologies and anoxic to oxic groundwaters

**Fig. 1:**
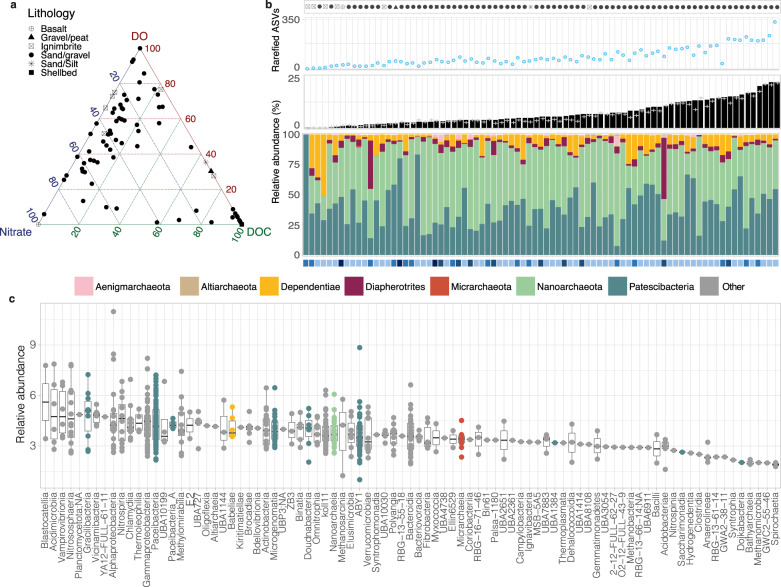
Distribution and abundance of ultra-small prokaryotes across groundwater sites. **a** Distribution of groundwater samples along DOC (0–26 g/m^3^), DO (0.37–7.5 g/m^3^) and nitrate-N (0.45–12.6 g/m^3^) concentrations scaled between 0 and 100. **b** Top plot: Richness of ultra-small prokaryote variants (rarefied ASVs) at each site. Middle plot: Proportion of ultra-small prokaryotes compared with the total microbial communities (black bars = OTUs, grey crosses = ASVs). Samples are ordered from least to most abundant. Lower plot: Phylum-level breakdown of amplicon based-relative abundance of Patescibacteria, Dependentiae and DPANN archaea (bottom). Symbol bars indicate aquifer lithology (top symbol bar), and oxygen content (lower symbol bar) with dark to light blue shading representing anoxic, suboxic, dysoxic to oxic groundwater. **c** Class-level rank abundance curve showing the average relative abundance of each genome across sites. The center line of each boxplot represents the median; the top and bottom lines are the first and third quartiles, respectively; and the whiskers show 1.5 times the interquartile range.

We used 16S rRNA gene amplicons to assess microbial community composition in 81 groundwater samples. Samples were collected from 59 wells over 10 aquifers in four geographic regions, separated by over a thousand kilometers, and encompassed wide-ranging aquifer chemistries and lithologies (Fig. 1a), comprising primarily shallow sandy-gravel aquifers, but also sand/silt, gravel/peat, volcanic (basalt, ignimbrite) and shell-bed aquifers (Table S1). A large portion of microbial community diversity comprised ultra-small groups of prokaryotes (Fig. 1b). Out of 52,553 OTUs, 21.8% (or 18.4% of 46,713 ASVs) were assigned to seven ultra-small microbial phyla (when considering CPR as the single Patescibacteria phylum). These comprised the bacterial phyla Patescibacteria and Dependentiae, and archaeal DPANN radiation. Altiarchaeota was included in DPANN as previously suggested [63, 64], although its taxonomic placement is uncertain due to genomic under-sampling [65, 66].

Ultra-small prokaryotes were detected in all samples, regardless of lithology, chemistry or geography. They have also been reported from several aquifers and lithologies in the USA (sandy gravel, agriculturally-impacted river sediment, mixed marine sedimentary/metasedimentary rocks, plutonic rock, and sandstone [10, 18, 19, 22], and from a carbonate rock aquifer system in Germany [67, 68]. Collectively these findings demonstrate that ultra-small microorganisms are geographically widespread across diverse aquifer lithologies. Moreover, while ultra-small microorganisms have mostly been detected in anoxic environments [69–72] or cultivated under anoxic conditions [15, 25], we found representatives in all oxic groundwaters (>3 mg/L DO) [73] (54/81 samples, Table S1). A few members of DPANN and Patescibacteria lineages have previously been detected in oxic environments [28, 67, 68, 74, 75], suggesting a degree of oxygen tolerance (genetic evidence presented below) or that these organisms are concentrated in anoxic niches within the aquifer substrate.

The relative abundance of ultra-small microorganisms was highly variable across the studied aquifers, ranging from 0.04% to 22% of all bacterial and archaeal 16S rRNA gene sequences (7.2% average ±5.5% standard deviation; Fig. 1b). Samples with low relative abundances of ultra-small microorganisms (lower than the average) had overall lower alpha diversity (Shannon diversity indices and OTU or ASV richness) and were mostly from volcanic aquifer sites (Fig. 1b; Table S2). At the phylum level, Patescibacteria and Nanoarchaeota tended to dominate groundwater ultra-small communities (Fig. 1b). However, we found that ultra-small species level diversity overall was considerable with up to 1429 unique OTUs in a single groundwater sample (or up to 653 variants via the more conservative ASV method) (Table S2). Rarefaction curves show most variant diversity was captured across all samples, with curve slopes equaling zero (or approaching zero post rarefaction) (Fig. S1; Table S2). Finally, our results confirm the site specificity of ultra-small prokaryotes [10], with only 16 OTUs common across ≥50% of all 81 groundwater samples, or five ASVs across ≥20% of samples (three Parcubacteria, a Ca. Uhrbacteria, and a Woesearchaeales) (Table S2).

### High shared phylogenetic and genomic similarity to ultra-small prokaryotes from groundwaters elsewhere

**Fig. 2:**
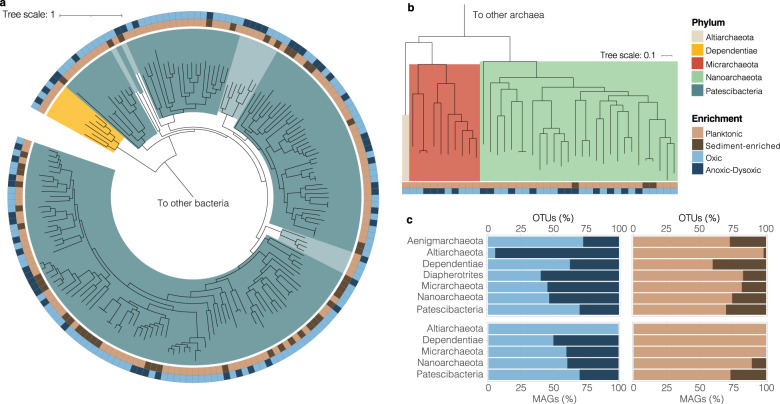
Diversity of groundwater ultra-small microbial communities. Maximum likelihood phylogenetic trees of 177 unique ultra-small bacterial MAGs (**a**) and 39 unique ultra-small archaeal MAGs (**b**) recovered in this study. Outer rings indicate the site characteristics where MAGs were enriched. Enrichment factors were calculated as (average relative abundance in oxic and planktonic ultra-small microbial communities, respectively)/(average relative abundance in anoxic-to-dysoxic or sediment-enriched microbial communities, respectively). Trees are based on either 120 concatenated bacterial marker genes or 122 concatenated archaeal marker genes from GTDB-Tk, and were rooted to other groundwater bacterial and archaeal MAGs, respectively (Table S3). Scale bars indicate the number of substitutions per site. Branch background shading denotes Patescibacteria classes (clockwise): Gracilibacteria, Saccharimonadia, UBA1384, Dojkabacteria, Microgenomatia, Doudnabacteria, ABY1, Paceibacteria_A and Paceibacteria. **c** Proportion of ultra-small microbial OTUs (top) and MAGs (bottom) enriched in low and high oxygen groundwater, and in planktonic and sediment-enriched samples (Table S1). Enrichment factors were calculated as described above.

**Fig. 3:**
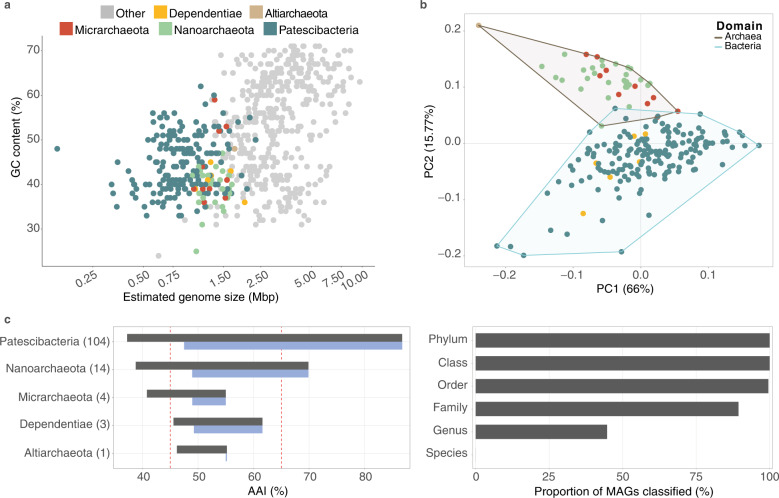
Estimated genome size, metabolic content and novelty of groundwater ultra-small prokaryotes. **a** Estimated genome size of groundwater MAGs calculated as (bin size – (bin size * contamination)) / (completeness), as described by Castelle et al. [9]. Genomes of ultra-small prokaryotes are colored by phylum-level. Other microbial genomes are shown in grey. **b** Principal Component Analysis (PCA) based on the composition of COG metabolic categories in recovered ultra-small MAGs. **c** (Right) Range of all pairwise AAI values (grey) and maximum AAI values (blue) between ultra-small prokaryote MAGs recovered in this study and GTDB representative genomes for a given phylum. Red dashed lines represent the AAI range defining the same family of organisms (45–65%) [74]. The number of genomes included in this analysis is indicated for each phylum in brackets. (Left) Proportion of ultra-small prokaryotic MAGs reconstructed in this study classified at each taxonomic level using GTDB-Tk.

To further assess the phylogeny and assess the genomic attributes and metabolic capacities of groundwater microbial communities, we reconstructed MAGs from 16 groundwater samples (eight wells over four sites and two aquifers). The dataset comprised 7,695 MAGs, including 539 unique MAGs (>50% complete, <5% contamination, dereplicated at 99% ANI) with 183 near complete genomes (>90% complete) (Table S3; Fig. S2). Based on phylogenetic analysis using GTDB [7, 76], MAGs represent 51 phyla, including five ultra-small microbial phyla (Table S3; Fig. S3). The ultra-small MAGs were found at all four sites and accounted for >1/3 of all unique MAGs (216 MAGs 50–100% complete, with 76 MAGs >90% complete). MAGs included 171 assigned to Patescibacteria, six to Dependentiae, and 39 to DPANN archaea (28 Nanoarchaeota, 10 Micrarchaeota, and one Altiarchaeota; Fig. 2a, b). The high representation of ultra-small prokaryotes in the MAG dataset further highlights the prevalence, diversity and abundance of these organisms in groundwater. Consistent with previous studies [6, 9, 77], genomes of ultra-small prokaryotes were small (1 ± 0.4 Mbp on average) with a tendency towards low GC contents (Figs. 3a, S2), and possessed limited metabolic capacities, which significantly differ between ultra-small bacterial and archaeal domains (results in Supplementary Materials; Figs. 3b, S2, S4).

Compared to reference genomes (GTDB species representatives), all recovered ultra-small MAGs are predicted to be novel species [78], and almost half were novel groundwater genera (Fig. 3c, results in Supplementary Materials). Most shared the highest affinity matches with other ultra-small genomes derived from aquifers elsewhere (e.g., in the USA), indicating niche adaptation within these lineages (although ultra-small MAGs from these groundwater ecosystems are over-represented in the GTDB database). Niche-specific phylogenetic conservation among geographically distant microorganisms in groundwater has likewise been reported among geographically distant anammox bacteria in groundwater [30].

### Ultra-small microbial communities were structured by geography, lithology, and dissolved oxygen concentrations

**Fig. 4:**
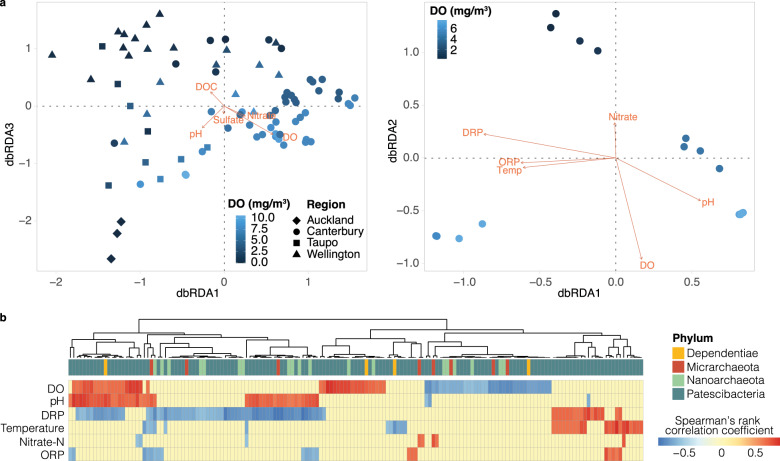
Influence of environmental variables on ultra-small microbial communities. **a** Distance-based Redundancy Analysis (dbRDA) of Bray-Curtis dissimilarities between groundwater communities based on 16S rRNA gene amplicon (left) and MAG relative abundance data (right). Samples are colored according to groundwater dissolved oxygen (DO) concentrations (mg/m^3^). The vectors indicate fitted environmental variables significantly correlated to dbRDA coordinates (permutation test, *p* < 0.05, permutations = 999). For amplicons, the categorical variables region and lithology were also significant (*p* = 0.001 each), while DRP, temperature and conductivity were not significant. For MAGs, sample type was not significant. Only variables measured for all samples and not significantly co-varying were tested. **b** Heatmap showing pairwise Spearman’s rank correlations between MAG relative abundance and environmental parameters significantly correlated with differences in ultra-small microbial communities based on dbRDA (right plot in a). Only significant Spearman’s rank correlations are shown for each MAG (*p* < 0.05). MAG phyla affiliations are indicated by the topmost bar.

While ultra-small prokaryotes were ubiquitous in groundwater, and overall highly similar to those found in groundwater at different global locations, community compositions varied across sites. To investigate environmental factors (Table S1) influencing ultra-small community composition, we performed distance-based redundancy analysis (Fig. 4a). DO, pH, nitrate-N, sulfate, and DOC were significantly associated with differences in the distribution of 16S rRNA gene amplicon sequences annotated as Patescibacteria, Dependentiae and DPANN (permutation test, *p* < 0.05). Additionally, lithology along with geographical region sampled significantly contributed to differences in ultra-small community composition (permutation test, *p* < 0.001). These parameters explained a much lower proportion (adjusted R-squared = 6.7%) of variance in the spatially broader amplicon dataset, compared with the smaller single-region metagenomic dataset. This is likely due to there being more aquifer types in the amplicon survey, and higher sequencing depth, recovering more of the rare ultra-small microbial biosphere. Instead, DO, ORP, temperature, pH, nitrate-N and DRP explained 73.1% (adjusted R-squared) of the variance of the Canterbury sandy-gravel aquifer metagenomic dataset.

Both amplicon- and genome-based ordinations showed clear separation of ultra-small communities along a progressive DO gradient (Fig. 4a). Although mostly described from anoxic environments and predicted to be fermenters [6, 9], some ultra-small organisms in this study were exclusively found at oxic sites, and a range of ultra-small organisms exhibited positive correlations (Spearman’s rank) between their respective relative abundances and dissolved oxygen concentrations (Fig. 4b). These results suggest some members of ultra-small phyla are adapted to oxic or partially oxic conditions. Given the rarity of complete or near complete respiratory chains [29], this is likely due to greater oxygen tolerance (Fig. S4). Oxygen tolerance could support partially anaerobic nitrogen metabolism [18, 29], aerobic fermentation, or the search for, and colonisation of, anoxic niches in oxic groundwaters. Bulk dissolved oxygen concentrations measured may not reflect the spatial heterogeneity of aquifers, and does not capture oxygen gradients present within biofilms (both likely to be present on suspended sediments sampled in groundwater and sediment-enriched groundwater fractions). Therefore, the high relative abundance of these organisms in oxic groundwaters (Fig. 1b) cannot be used as an independent indicator of aerobic lifestyles.

### Evidence for both anaerobic and oxygen-tolerant lifestyles

The results indicated groundwater oxygen content plays an important role in structuring ultra-small communities (Fig. 4). Overall, ultra-small prokaryotes also showed a preference for oxic conditions (70% of Patescibacteria MAGs, 50% of Dependentiae, 61% of Nanoarchaeota, 60% of Micrarchaeota and of 100% Altiarchaeota were enriched in oxygen-rich samples; Fig. 2c; Table S4). We therefore investigated the distribution of metabolic functions linked to groundwater redox categories, and potential oxygen-dependent adaptations. Results showed no major differences in metabolic potential between oxic versus dysoxic ultra-small populations, except cell motility related genes were more frequently detected in ultra-small prokaryotes associated with low oxygen (dysoxic) groundwaters (Table S2; 2.4 genes ± 2.2 SD per Mbp for dysoxic-enriched MAGs and 1.3 genes ± 1.7 SD per Mbp for oxic-enriched MAGs; *p* < 0.01, Wilcoxon signed rank test). These included genes encoding type II/IV secretion system proteins in DPANN archaea (i.e., components of archaeal flagella) and the toxin-antitoxin system, HicAB, which is suggested to regulate the formation of persister cells in bacteria [79]. These mechanisms may indicate a greater need to respond to stress, or locate new hosts or resources in low oxygen groundwaters. However, the overall similarity of metabolic profiles between these two environments leads us to suggest that ultra-small prokaryotes possess the genomic flexibility to readily adapt to changes in oxygen concentrations in aquifers. A wide range of ecological niches could also imply flexibility in host range, as recently suggested for DPANN archaea [80].

Regardless of niche-preference, results showed that ultra-small microorganisms are genomically equipped to degrade complex organic carbon molecules via fermentation (58 MAGs) and protein hydrolysis (53 MAGs) (Fig. S4), as for genomes described from US aquifers [18, 69]. The detection of nitrite reductase genes (*nirK*) in 11 genomes highlights their proposed contribution to groundwater nitrogen cycling [9, 18] via the reduction of nitrite to nitric oxide, and use of nitrite as an alternative electron acceptor. Additionally, the genomes lacked genes involved in the majority of the oxidative phosphorylation pathway. In contrast, components of ATP synthase/complex V were nearly consistently present. This may represent a key mechanism by which these organisms generate energy after scavenging protons from their host [9]. Along with the presence of fermentation genes (for example, L-lactate dehydrogenase), these observations are consistent with an anaerobic lifestyle [9, 23].

The paradoxically high relative abundance of Patescibacteria, Dependentiae, and DPANN archaea in partially or fully oxic groundwater could be explained by the widespread presence of genes involved in antioxidant systems, such as superoxide dismutase, thioredoxin, and glutathione peroxidase genes (observed in 144 MAGs, Table S5). This implies an adaptation in these predicted fermenters to (micro)aerobic environments by protecting against reactive oxygen species, as found for example in Saccharibacteria (class Saccharimonadia in GTDB) in soil [81]. In addition, five Patescibacteria MAGs contained genes encoding subunits of cytochrome o ubiquinol oxidase (complex IV of the respiratory chain, involved in aerobic metabolism). This complex was previously described in Patescibacteria recovered from oxic environments and is suggested to be used for detoxification rather than energy production because of the absence of other components of the respiratory chain [6, 28, 29]. Results therefore provide additional evidence for aerotolerance among ultra-small phyla.

### Groundwater oxygen content shapes ultra-small cell transcriptional activity

Although genes for antioxidant systems were widespread, and were found in the genomes of ultra-small prokaryotes enriched in both oxic and dysoxic conditions, transcripts for seven antioxidant genes (four superoxide dismutase, three thioredoxin) were detected exclusively in dysoxic groundwater samples (Table S5). This suggests they represent a mechanism for tolerating low levels of oxygen in these samples or facultative anaerobic metabolism. Mechanisms for oxygen tolerance employed in oxic conditions require further investigation, such as identifying relatively lower levels of potential antioxidant gene expression. However, transcript expression in oxic groundwaters was not overall lower. A greater portion of community transcriptional activity derived from ultra-small taxa in oxic groundwater (Table S6). Consistent with differences in ultra-small community composition between dysoxic and oxic sites, the activity of some of these organisms was also observed only in oxic groundwater. Transcripts from 28 ultra-small bacteria and archaea were exclusively detected in dysoxic samples, while nine ultra-small prokaryotes were exclusively active in oxic samples. Differences were even stronger at gene-level expression, with 4669 transcripts exclusively detected in dysoxic and 3235 in oxic samples (Table S7), representing 54% and 38% of all expressed genes, respectively.

**Fig. 5:**
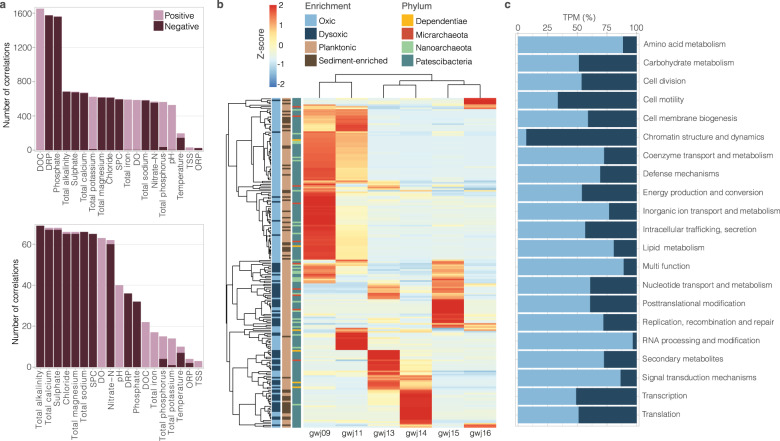
Ultra-small transcriptional profiles across samples and relationship to groundwater physicochemical parameters. **a** Number of significant Pearson correlations between modified-TPM values and groundwater parameters at gene (top) and genome (bottom) levels (*p* < 0.05). Pearson correlation coefficients range from 0.81 to 0.99 and −0.99 to −0.81 for both gene level and species level correlations. **b** Heatmap showing total modified-TPM per MAG across six groundwater metatranscriptomes. modified-TPM values were scaled to row and column (*Z*-score). **c** Distribution of ultra-small prokaryote modified-TPM across EggNOG metabolic categories in oxic and dysoxic groundwater samples. Proportions were based on average total modified-TPM per metabolic category across oxic vs. dysoxic sites.

When considering genes encoding functions other than antioxidants, we found that the expression of genes involved in amino acid metabolism (88% of total modified-TPM for the metabolic category), signal transduction (86%) and lipid metabolism (81%) were more prevalent in oxic groundwater. Dysoxic groundwaters hosted higher activity related to chromatin structure (93%) and cell motility (66%), including genes encoding elements of archaeal flagella (type II secretion system like), which were preferentially expressed in dysoxic samples (Table S7). Greater motility could indicate an increased demand by some archaea and bacteria to search for resources in the dysoxic groundwater sampled. Oxic and dysoxic environments were therefore demarcated by large differences in overall gene expression. Ultra-small transcriptional activity was positively correlated with DO, along with DOC, total iron and pH, and negatively correlated with phosphate, sulfate, and the cations calcium, magnesium and sodium (Pearson correlation coefficients, *p* < 0.05; Fig. 5a), at both gene (all expressed genes) and genome levels. DO was linked to the highest number of positive correlations at the genome level, and gene expression per ultra-small taxa was on average 4 to 18-fold higher in oxic versus dysoxic samples (Table S6). The trend was comparable, but less pronounced, for other groundwater microorganisms (0.8 to 3.7-fold change in average modified-TPM per MAG in oxic compared to dysoxic samples; Table S6). While metabolic capacities differed little between oxic- and dysoxic-enriched ultra-small prokaryotes, genome-level transcriptional profiles showed clear separation between groundwater oxygenation regimes (Figs. 5b, S5). A common feature of these organisms could therefore be the ability to colonise a broad redox spectrum and cope with fluctuating redox conditions.

### Genetic differentiation of planktonic and sediment-attached ultra-small populations

**Fig. 6:**
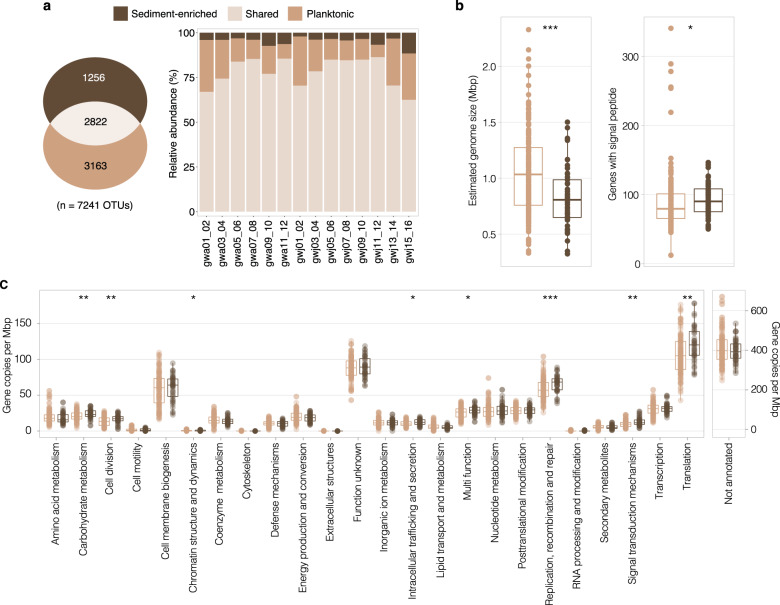
Planktonic and sediment-enriched ultra-small microbial fractions in aquifers. **a** Venn diagram comparing planktonic and sediment-attached ultra-small microbial communities based on the presence/absence of related 16S rRNA OTUs (left) and relative abundance of each fraction (right). **b** Difference in estimated genome size and genes containing predicted signal peptides between ultra-small MAGs enriched in the planktonic and sediment-enriched aquifer fractions. **c** Gene copy numbers across metabolism in planktonic and sediment-enriched associated ultra-small MAGs. Copy numbers were normalized to estimated genome size. Meaning of box-plot elements described above. Statistical significance was assessed using Wilcoxon Signed Rank (*p < 0.05, **p < 0.01; ***p < 0.001).

Both groundwater and sediment-enriched groundwater were collected in this study. Detachment of biofilms or biofilm-coated aquifer particles, leading to groundwater biomass enrichment, was confirmed by higher heterotrophic plate count values, with a median increase of 17-fold in sediment-enriched samples (Table S8), indicating an increase in the viable microbial biomass collected following in situ sonication. This is in line with results indicating aquifer sediments harbor up to one to two magnitude greater microbial biomass than groundwater [82]. Planktonic and sediment-attached microbial fractions are reported to only share one third of taxa [83]. Here, results demonstrated this trend for ultra-small communities, showing 2,822 OTUs (39%) were shared between the two fractions (Fig. 6a). We identified a unique population of ultra-small microorganisms in the sediment-enriched aquifer fraction (17% of OTUs). However, these OTUs represented only 2.1–11.6% of the ultra-small microbial communities in terms of relative abundance (Fig. 6a).

An analysis of MAG relative abundance profiles showed that 27% of Patescibacteria (particularly Saccharimonadia and Dodjkabacteria classes) and 11% of Nanoarchaeota were generally more abundant in sediment-enriched groundwater, while all Dependentiae, Micrarchaeota and Altiarchaeota MAGs were exclusively enriched in the planktonic fraction (Fig. 2c). Ultra-small prokaryotes in the sediment-enriched fraction had significantly smaller genomes than their planktonic counterparts (Fig. 6b; *p* < 0.001, Wilcoxon signed rank test). Smaller genome sizes in free-living bacteria are associated with high environmental stability, high resource competition and scarce nutrients [84, 85]. We hypothesize that the more reduced genomes among the attached ultra-small fraction is due to greater niche stability and more stable interspecies (e.g. host-symbiont) interactions.

We then searched for metabolic adaptation to surface-associated lifestyle between sediment-attached and planktonic ultra-small MAGs. When considering differences in estimated genome size, gene copy numbers (per Mbp) for different metabolic pathways differed significantly between the two populations (Fig. 6c). Those encoding functions that play a role in biofilm formation and maintenance, such as signal transduction or secretion, were more frequent in sediment-enriched ultra-small genomes. For example, genes encoding proteins resembling CheY, which is a response regulator for bacterial chemotaxis and adhesion [86], were twice as abundant in sediment-enriched MAGs (2.1-fold more copies in sediment-enriched vs planktonic MAGs). Additionally, genes predicted to encode signal peptides were more frequent in MAGs associated with the sediment-enriched fraction (Fig. 6b, Wilcoxon signed rank test, *p* < 0.05), indicating a greater reliance on periplasmic or extracellular proteins. Of these, we found 1.2-fold more genes related to Sec translocation complex (e.g. *secA,D,E,F,G,Y*), and 1.7-fold more genes encoding diguanylate cyclases. These latter enzymes synthesize cyclic di-GMP, a widespread signaling molecule promoting bacterial biofilm formation [87]. Results therefore reveal genetic differentiation between attached and planktonic ultra-small populations linked to biofilm formation.

**Fig. 7:**
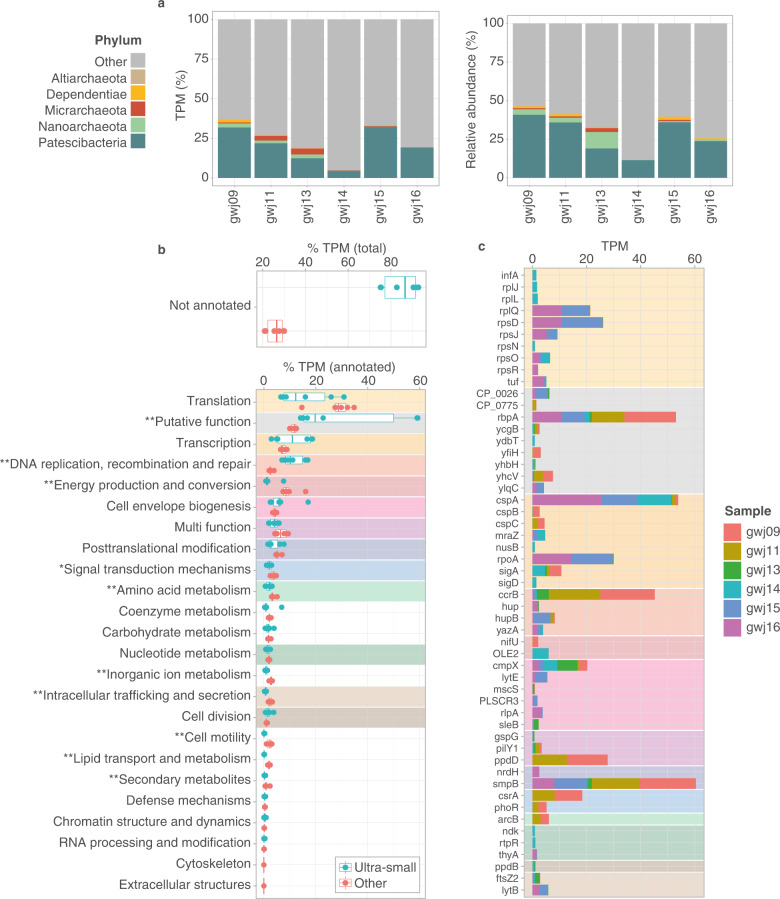
Gene expression profiles of ultra-small microorganisms. **a** Proportion of total modified-TPM expressed by ultra-small prokaryotes (left). Modified-TPM was normalized based on the genome size of respective MAGs. Genome relative abundance of ultra-small prokaryote MAGs compared to the rest of the communities (right). Taxonomy is indicated at phylum level. Groundwater for samples gwj09,11 was oxic with 0–0.8 g/m^3^ DOC, and for gwj13–16 was dysoxic with 3.1–26 g/m^3^ DOC. **b** Proportion of expressed genes not assigned (“Not annotated”, upper plot) or assigned (lower plot) to COG functional categories in ultra-small prokaryotes and other groundwater microorganisms. The center line of each boxplot represents the median; the top and bottom lines are the first and third quartiles, respectively; and the whiskers show 1.5 times the interquartile range. Significant differences were assessed for each function using Wilcoxon Signed Rank (**p < 0.01, *p < 0.05). Meaning of box-plot elements described above. **c** Expression of the top 20 genes annotated with a gene name across samples. Expression of *cspA,B,C* genes (cold shock proteins) is likely due to the sample preservation method used. Plot background colors denote the same functional categories in (**b**) and (**c**). The grey background shading indicates genes in the EggNOG ‘Function unknown’ category, which are assigned a putative function based on sequence orthology with poorly characterized genes.

Of the top 30 differentially expressed genes by sediment-enriched and planktonic ultra-small lineages (with known metabolic function), the planktonic fraction more highly expressed genes involved in energy production and housekeeping functions (e.g., amino acid metabolism and DNA replication), while the sediment-enriched fraction more highly expressed membrane-related genes (Table S7). The latter included mainly poorly characterized genes (*OLE2*, *ydbT*, *hldE*, *pmp15*, *MA20_31645*, *algI*, *fmt*, *leuA*, *yebC*, *neuA*), of which proposed functions include the regulation of exopolysaccharides (*yebC*) [88] and the synthesis of precursors used in lipopolysaccharide biosynthesis (*hldE*) [89]. We detected a smaller total number of expressed genes encoding signal peptides in sediment-enriched (180 and 422 genes in gwj14 and gwj16 respectively, compared to 419 and 550 genes in gwj13 and gwj15). This was likely due to the smaller predicted genome size of ultra-small MAGs enriched in the attached compared to planktonic groundwater fraction (Fig. 7b), and a smaller number of taxa associated with aquifer particles (49 vs. 167 MAGs respectively). However, these genes were expressed at a similar level (total modified-TPM in gwj16 was 0.95% of gwj15) or were more active (1.4-fold higher in gwj14 than gwj13). In addition to differences in genomic potentials described above, gene expression profiles further suggest specific adaptations of ultra-small prokaryotes for biofilms, as shown previously in host-associated microorganisms [90, 91].

### Compositional similarity among sampled size fractions in planktonic and sediment-associated communities

The average diameter of Patescibacteria and DPANN is 0.2 μm [92]. It is predicted that the capture of ultra-small prokaryotes on larger pore size filters is due to their active involvement in host-symbiont associations, and that cells passing through this pore size are ‘unattached’ [10], whether due to sampling disruption or as part of a natural free-living state. Conversely, the >0.22 μm fraction would include unattached cells larger than the average, and is also likely to include smaller cells trapped due to pores being clogged by other cells or particles, and cells in particle-attached biofilms. The ultra-small communities analyzed here are inferred to have constituted a mixed fraction that includes the host-attached fraction, although further studies are needed for validation. The >0.2 μm ultra-small fraction in groundwater, in terms of absolute biomass, is reportedly substantial, exceeding cells captured in the 0.1–0.2 μm pore size range [10]. To determine the putative ‘unattached’ fraction that may be missed by typical filtration approaches, including 0.1 μm pore size filtration, we undertook a broad-spectrum size fraction analysis on groundwater from well E1, on samples collected before and after in situ sonication, including tangential flow filtration (>1.5 nm fraction) along with other size fractions captured using standard direct flow filter membranes (0.1–0.22 μm, 0.22–1.2 μm, >0.22 μm, >1.2 μm) (results in Supplementary Materials; Fig. S6). Consistent with previous findings [10] a substantial fraction of ultra-small taxa (10,454/10,981 OTUs or 9,165 exclusively) was captured by the >0.22 μm filtration method (i.e. the putative host-attached and mixed fraction) versus 597/10,981 OTUs captured by the 0.1–0.22 μm solely unattached fraction method, or 78 OTUs exclusively. We also show that the ultra-small community fraction is highest for each filter fraction before sonication (Fig. S6, and the ultra-small fraction is on average 2.3 times higher in groundwater before sonication when considering all 14 > 2 μm pairs), indicating that cells liberated from aquifer biofilms contained a greater fraction of ‘other’ taxa, and that these other taxa encompass a range of cell sizes overlapping with the DPANN and Patescibacteria lineages (as indicated in Fig. 3). Although ultra-small taxa enrichment biases exist between 0.1–0.2 μm and very large (+2.5 μm) filter fractions [10], when comparing each size fraction sampled here, we found that well E1 ultra-small community fractions clustered tightly together relative to other wells (based on an ordination of Bray-Curtis dissimilarities), with the only clear difference being between groundwater and sediment-enriched samples. While further analyses are needed across a distribution of groundwater sites and physicochemistries to extend findings, results here indicate little difference in ultra-small community compositions among the different size fractions (results in Supplementary Materials; Fig. S6), and hence among cells that are purportedly host-attached or unattached.

### Ultra-small microorganisms are major contributors to community gene expression, but most is of unknown function

Due to their small cell and genome sizes, it has been questioned whether ultra-small prokaryotes are cellular life forms rather than extrachromosomal DNA molecules within host cells or virus-like organisms [9]. However, they possess characteristic cellular machinery, including that necessary for protein synthesis and cellular division [9], and microscopy evidence indicates they have typical prokaryotic cell and membrane morphologies [10, 19]. Moreover, the fermentative activity of a small group of ultra-small bacteria has been illustrated via proteomics, following aquifer acetate-amendment [69]. Here, using metatranscriptomes from six groundwater samples, we show that they contribute a substantial portion of gene expression in natural aquifer microbial communities. Levels of gene transcripts from Patescibacteria, Dependentiae and DPANN organisms (except Altiarchaeota for which no transcripts were recovered) were relatively high in groundwater, ranging between 5% and 37% of gene expression (normalized to estimated genome size) across sites, and mirroring the relative abundance of these taxa (5–46% of total genome coverage) (Fig. 7a). The ratio of genome relative abundances to gene expression was within the same range as other groundwater prokaryotes, albeit at the higher end for ultra-small taxa (Fig. S5). Results temper previous research suggesting that a limited protein biosynthesis machinery in these organisms could limit transcript abundance [64].

Nonetheless, 75–93% of the overall gene expression by ultra-small prokaryotes could not be linked to any functional category based on EggNOG orthogroup assignment (compared to 21–30% for the rest of the communities) (Fig. 7b). The vast majority of highly expressed genes across sites lacked predicted functions, an observation consistent with results from metatranscriptomes in the deep subsurface [27]. Coupled with the lack of vital biosynthetic capacities of ultra-small microorganisms, and the complexity of groundwater microbial communities, these results further highlight the challenges, and the need, for cultivating representatives.

### Metabolic expression by ultra-small microorganisms is consistent with genome streamlining and symbiotic lifestyles

While the functional contributions of a sizeable fraction of gene transcripts is unknown, ultra-small organisms had distinct transcriptional profiles compared to other groundwater microorganisms (Fig. 7, S5), consistent with genome-streamlining, their predicted symbiotic lifestyles and auxotrophies. When solely analyzing the expression of annotated genes, metabolic functions such as energy production, lipid and amino acid biosynthesis, were significantly less active in ultra-small prokaryotes (Fig. 7b, *p* < 0.05, Wilcoxon signed-rank test). These results are in accordance with the absence of numerous metabolic pathways, including the respiratory chain and fatty acid biosynthesis (Fig. S4), and their predicted symbiotic role [6, 9]. For example, members of the Patescibacteria are thought to uptake lipids from their hosts [93], although we detected no evidence of activity associated with lipid transport (i.e. lipid transporter or translocase gene transcripts). Less transcriptional investment in secondary metabolites is also consistent with genome streamlining, and the reduced presence of their encoding in Patescibacteria and DPANN genomes [92]. Moreover, relatively low levels of cell motility, with the exception of cell-to-cell attachment mechanisms (see section below), supports evidence that these organisms form stable physical associations with hosts [22, 94, 95]. Instead, ultra-small microorganisms collectively invested most of their annotatable transcriptional effort into protein biosynthesis (transcription and translation), and at levels similar to other members of the groundwater communities (Fig. 7b). For example, numerous genes encoding ribosomal protein subunits (204 small and 215 large subunit genes, e.g. *rplQ* and *rpsD*) were expressed by these organisms across all sites. These were among the 20 most highly expressed annotated genes by ultra-small microorganisms overall (Fig. 7c). While annotated gene transcripts may encode a greater portion of core functions, relatively high expression of ribosomal protein encoding genes is expected given the universal distribution of these highly conserved genes across bacteria and archaea, along with their central role in cellular protein production.

Among other highly expressed genes detected were those involved in conserved functions across bacterial and archaeal domains. These included the *csrA* carbon storage regulator involved in post-translation regulation, *smpB* in the ribosome rescue system, and the DNA-directed RNA polymerase alpha subunit *rpoA* involved in transcription (Fig. 7c). However, data indicated that ultra-small prokaryotes were significantly more active in DNA replication, recombination and repair than other groundwater prokaryotes (Fig. 7b). In particular, the recombinase *ccrB* gene, shown to be involved in genomic island excision/integration in pathogenic bacteria [96] was highly expressed by two Doudnabacteria and one Paceibacteria. An additional 14 MAGs expressed a gene with a similar recombinase domain. This could indicate plasticity in the genomes of these ultra-small prokaryotes and a mechanism to increase their genetic diversity, in addition to diversity-generating retroelements (DGRs) present in these organisms [97].

Patescibacteria are genomically inferred to have slow genome replication rates [10, 98], equivalent to a fraction of each population replicating at any given time. However, estimated rates are similar to those observed for other groundwater prokaryotes [10], and even broadly similar to those for bacteria colonising human guts [98]. As for these other groups of organisms, patescibacterial populations can also replicate faster with at least every member replicating at a given time. Consistent with active replication, we found that members from all ultra-small phyla, including six of the nine classes of Patescibacteria identified (ABY1, Doudnabacteria, Gracilibacteria, Microgenomatia, Paceibacteria, Paceibacteria_A), expressed genes involved in cell division (Table S5). The results indicated they were actively dividing to sustain populations within geochemically diverse aquifers (including oxic to dysoxic and DOC poor to DOC-rich groundwater), and that the transcriptional investment in cell division was equivalent to other groundwater prokaryotes (Fig. 7b). The gene encoding the cell division protein FtsZ2 was amongst the most highly expressed annotated genes (Fig. 7c). We also detected an additional 10 cell division genes (*ftsA*,*E*,*H*,*I*,*K*,*W*,*X*,*Y*,*Z*,*Z1*) expressed by 59 of these organisms across all four groundwater wells from which transcriptomes were obtained.

### Presence of pili and competence related genes and transcripts

Ultra-small genomes encoded numerous, prevalent competence related genes (*comEC, dprA*) as well as type IV-pilin-like genes, which are involved in the uptake of eDNA molecules and adherence in cell-to-cell interactions [99]. Results confirm previous microscopic observations of pili-harboring ultra-small cells in groundwater [10, 100]. Evidence for pili production was further indicated by the detection of transcripts of type IV pili genes, *pilY1* and *ppdD*, across the four wells (Fig. 7c), along with the presence of *pilA,B,C,L,M,T,V,W* genes. These observations are in-line with microscopy evidence presented by He et al. [10], showing that the cell-to-cell interaction of ultra-small prokaryotes and host cells is mediated via pili structures, and together with our observed expression of numerous cell division genes, support their predictions that cell replication of CPR and DPANN episymbionts is stimulated by host attachment.

### Consistent associations among ultra-small community members across sites

**Fig. 8:**
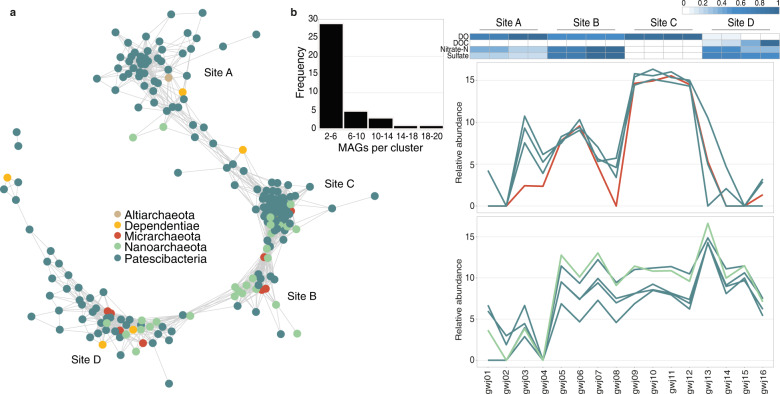
Co-occurrence of ultra-small prokaryotes across the 16 genomically-characterized groundwater samples. **a** Co-occurrence network of ultra-small microorganisms constructed based on relative genome abundance data and Spearman’s rank correlation coefficients. Only significant positive correlations are displayed (*ρ* > 0.8, *p*-value < 0.001). **b** Frequency of MAGs per cohort (left). Example of highly correlated genome relative abundance profiles of two ultra-small prokaryote cohorts across 16 groundwater samples (right). Cohorts were identified from genome relative abundance profiles using hierarchical clustering (“ward.D2” method) and confirmed using Spearman’s rank correlation analysis. Chemical parameters were scaled between 0 and 1.

Aquifer microbial communities have been shown to form spatial cohorts [21]. To examine interconnections between ultra-small taxa and cross-validate site-specific diversity, we compared the relative genome abundance profiles of ultra-small MAGs. Groups of phylogenetically diverse MAGs were consistently associated across sites, with co-varying abundances of Patescibacteria, Dependentiae and DPANN showing strong positive correlations (Spearman’s rank correlation coefficient *ρ* > 0.8, *p* < 0.001) (Fig. 8a). A co-occurrence network analysis, comprising 206 nodes (organisms) linked by 2439 edges (positive correlations), indicated four major clusters, corresponding to the four sampling sites. This demonstrates that each groundwater site (encompassing duplicate wells located meters apart and groundwater ± sediment-enriched fractions) contained a unique pattern of these organisms, reflecting the spatial heterogeneity shown by our amplicon-based findings and a recent study of US aquifers [10]. The four sites had contrasting groundwater geochemical profiles (Table S1), including oxic to dysoxic (0.37–7.5 g/m^3^ DO), uncontaminated to contaminated (0.45–12.6 g/m^3^ nitrate-N), and DOC-poor to relatively DOC-rich (0–26 g/m^3^) [101]. Clusters most likely delineate group preferences for particular groundwater conditions, based on shared genetic adaptations or host populations.

Using hierarchical clustering we further identified 39 cohorts of ultra-small bacteria and archaea (2–20 members/cohort) co-occurring across the groundwater samples (Fig. 8b, S7), representing similarly niche-adapted aquifer populations [21]. Members share similar preferences for aquifer fraction, namely planktonic or sediment-enriched (all members enriched in the same fraction in 28/39 cohorts), or for DO concentration (36/39 cohorts). Very strong spatial relative abundance patterns have been observed between subsurface hosts and episymbionts elsewhere [22, 28, 94], and amongst ultra-small prokaryotes [68, 102]. Here, the majority of recovered ultra-small taxa (206/216 MAGs) comprised cohorts. Our results indicate the extent to which ultra-small bacteria and archaea form spatial cohorts in aquifers.

## Conclusions

Prior and current research make it increasingly apparent that the ultra-small microbial phyla Patescibacteria (CPR), Dependentiae and DPANN are prevalent, and often abundant members of microbial communities, in geographically widespread aquifers. Results here showed a high level of phylogenetic conservation with ultra-small prokaryotes from aquifers elsewhere (mostly from the USA), with most genomes belonging to existing families, although exhibiting substantial novelty at genus and species levels. Results confirm these newly recovered species have reduced genome sizes, exhibit numerous auxotrophies, and could contribute to groundwater nitrogen and carbon biogeochemical cycles. We demonstrate that their community composition is strongly influenced by groundwater oxygen content and differentiated by groundwater fraction (attached or planktonic), and that ultra-small prokaryotes form cohorts with shared environmental preferences. Ultra-small prokaryotes exhibited high genomic flexibility in regard to oxygenation regimes, but specific metabolic adaptations related to biofilm formation and maintenance were more prevalent among sediment-associated versus planktonic taxa. Data further indicate ultra-small prokaryotes are equivalent to other groundwater prokaryotes in transcriptional and translational activity, but invest significantly less resources into energy and lipid production and motility. Findings highlight the need for metabolic function assignments for the majority of genes expressed by ultra-small microorganisms to better resolve their role in groundwater ecosystems.

## Supplementary information

Supplementary Materials

Supplementary Tables

## Data Availability

Sequence data is deposited with NCBI under BioProject PRJNA699054.
