# Supplementary material for: Ultra-small bacteria and archaea exhibit genetic flexibility towards groundwater oxygen content, and adaptations for attached or planktonic lifestyles
Source: ISME Commun. 2023 Feb 17;3:13. doi: 10.1038/s43705-023-00223-x (PMC9938205; doi:10.1038/s43705-023-00223-x)
Supplement: Supplementary file 1 — Supplementary Materials [file 43705_2023_223_MOESM1_ESM.pdf]

Supplementary Materials *(Methods, Results, Figures)*

Gios et al., Ultra-small bacteria and archaea exhibit genetic flexibility towards groundwater oxygen content, and adaptations for attached or planktonic lifestyles.

Supplementary Methods

Groundwater geochemistry

Dissolved oxygen (DO), water temperature, pH, specific conductivity (SPC) and redox potential (ORP) were measured on site using field probes (YSI EXO2 Sonde, Pro Plus and ProDSS, Yellow Springs, Ohio, USA). Concentrations of nitrite and nitrate were measured according to APHA 4500-NO_3 [1] at Hill Laboratories (Hamilton, NZ). Dissolved organic carbon (DOC) and sulfate concentrations were measured according to APHA 5310 C and APHA 4110 B, respectively [1]. Total phosphorus and phosphate were determined according to APHA 4500-P B and E, and dissolved reactive phosphorus (DRP) according to APHA 4500-P G [1]. Total alkalinity was measured according to APHA 2320 B [1] and chloride concentration was determined using APHA 4110 B [1]. Concentrations of total calcium, potassium, magnesium, iron and sodium were measured according to APHA 3125 B [1]. Total suspended solids (TSS) were measured using an in-house protocol. Briefly, groundwater samples were left to evaporate in an oven at 105 °C until dry. The weight of the dried solids was then measured and normalized to the volume of water analyzed.

Nucleic acid extraction

To remove RNAlater, filters were centrifuged (2500 g for 5 min) and washed with nuclease-free Phosphate-Buffered Saline (pH 7.4, ThermoFisher Scientific, Waltham, MA, USA). DNA was extracted using the DNeasy PowerSoil Pro Kit (Qiagen, Valencia, CA, USA) and 0.25-0.9 g of whole filter per reaction. Samples were extracted in replicate (1–47 reactions/sample). Replicates were pooled and concentrated using a sodium acetate/ethanol precipitation with glycogen (0.1 µg/µL final concentration, Roche, Basel, Switzerland). High molecular weight DNA was confirmed via gel electrophoresis. DNA was quantified using Qubit fluorometry (ThermoFisher Scientific), and quality was checked using a NanoPhotometer (Implen, Munich, Germany). Extractions yielded <0.010–8.12 ng/µl for 16S rRNA gene amplicon sequencing, and 70–864 ng of DNA (8.7 ng for gwj02) for metagenomics.

RNA was extracted from six Canterbury samples (gwj09, gwj11, gwj13-16; 1-3 reactions/sample) using the RNeasy PowerSoil Total RNA Kit (Qiagen). The final precipitation step was carried out overnight at -80°C with nuclease-free glycogen (0.1 μg/μL final concentration). Replicate extractions were pooled and purified using the RNA Clean & Concentrator-5 Kit (Zymo Research, Irvine, CA, USA). RNA quality and concentration were determined using Agilent RNA 6000 Pico chips and Bioanalyzer (Agilent, Santa Clara, CA, USA). Extractions yielded 0.630–140 ng RNA. RINs were 5.7–8.0 (excluding one sample with RIN=2, but DV200=98%, indicating almost all fragments were >200 bp).

Some samples or sample replicates yielded low or undetectable amounts of DNA/RNA, and served as negative control for filters. All samples yielding sufficient nucleic acid for analysis had microbial community compositions that are consistent with other groundwater studies (Fig. 1c).

16S rRNA gene amplification, sequencing, and processing

PCR amplification of 16S rRNA genes used modified 515F [2] and 806R [3] Earth Microbiome Project primers, with Illumina Nextera adapters. Reactions used 10 μl of template (initial DNA concentrations of <10 pg/μl to 8.12 ng/μl) and were performed using MyTaq™ HS Red Mix (Bioline, London, UK) as follows: initial denaturation of 95°C for 5 min, then 95°C for 45 s, 50°C for 60 s, 72°C for 90 s for 35 cycles, followed by a final extension of 10 min at 72°C. PCR product length was checked by agarose gel electrophoresis. Products were purified using Agencourt AMPure XP magnetic beads (Beckman Coulter, Brea, CA, USA) and quantified using Qubit fluorometry. Negative controls were included for 16S rRNA gene amplification but not sequenced, as no bands were visualized by gel electrophoresis. Libraries were prepared following manufacturer's instructions for 2x250 bp sequencing via the Illumina MiSeq platform with V2 chemistry at Auckland Genomics (University of Auckland, Auckland, NZ). For OTU generation, forward and reverse reads were merged using USEARCH v9.0.2132 [4]. Merged reads were filtered using sickle v01.33 (-q 30 -l 200, https://github.com/najoshi/sickle). Another 10 bp from each end was removed using USEARCH -fastx_truncate [4]. Sequences were dereplicated and clustered at 97% similarity using UPARSE [5], with chimera and singleton removal prior to generating Operational Taxonomic Units (OTUs). OTUs were classified using USEARCH -sintax

and the SILVA 132 database [6]. The OTU table was rarefied to 13,393 sequences per sample using QIIME2 v2018.2 [7], or 10,301 sequences per sample for the size fraction analysis. For amplicon sequence variant (ASV) generation, unmerged reads were processed using QIIME2 (2022.2) with the dada2 denoise-paired command and 10 bp removal from the start of reads and truncation set to 230 bp [7]. The ASV table was classified using the feature-classifier classify-sklearn command and the QIIME2 pre-trained classifier silva-138-99-515-806-nb-classifier.qza derived from the SILVA 138 database [6]. Taxonomic classifications were added to the ASV table and converted to a text file using biom add-metadata and convert commands. In R, non-prokaryotic sequences were removed, leaving 44,276 out of an initial 46,713 ASVs, and the data was rarefied to the minimum sample depth (3,104) with the phyloseq rarefy_even_depth command (bioconductor 3.16) [8]. Rarefaction curves of unrarefied Patescibacteria and DPANN ASVs were built using the vegan 2.6.4 rarecurve command with a step size of 50 [9].

Metagenome sequencing

Whole genome shotgun sequencing was undertaken on 16 Canterbury samples (gwj01-16) (Table S1). DNA libraries were prepared using the Illumina TruSeq Nano DNA Kit with ~550 bp inserts, by Otago Genomics (University of Otago, Dunedin, NZ). The low-yield gwj02 sample was prepared with the ThruPLEX DNA-Seq Kit (Takara Bio USA, Inc., Mountain View, CA, USA). Sequences (2×250 bp) were generated using the Illumina HiSeq 2500 V4 platform. Adapter sequences were removed using Cutadapt v2.10 [10], and reads were quality trimmed using sickle v01.33 (parameters -q 30 -l 80; https://github.com/najoshi/sickle). Read quality was checked using FastQC v0.11.7 (https://www.bioinformatics.babraham.ac.uk/projects/fastqc/).

Metagenome assembly and binning

The 16 metagenomes were individually assembled using SPAdes v3.11.1 [11] (parameters: --meta, -k 43,55,77,99,121). Metagenomes from the same well also were co-assembled (same parameters). Reads were mapped to scaffolds to recover coverage per metagenome using Bowtie v1.2.0 [12] (-n 1 -l 222 --minins 200 --maxins 800 –best). Scaffolds (≥2kb long) were binned using MetaBAT2 v2.12.1 [13], MaxBin

v2.2.6 [14] and CONCOCT [15], with default settings, including differential coverage information. A non-redundant set of bins was selected for each assembly with DAS_Tool v1.1.1 [16]. Bins were dereplicated across all assemblies using dRep v2.0.5 [17] (99% average nucleotide identity, ANI, threshold). Bins (metagenome-assembled genomes, MAGs) were manually refined by performing a *t*-distributed Stochastic Neighbor Embedding (*t-SNE*) transformation using tetranucleotide frequency and coverage values (https://github.com/dwwaite/bin_detangling) and visualization with VizBin [18].

RNA sequencing and metatranscriptome analysis

RNA libraries were prepared by Otago Genomics using the Ovation SoLo RNA-Seq System (NuGEN, Redwood City, CA, USA). Genomic DNA was removed as a part of the library preparation kit using a DNase treatment. To remove rRNA from the RNA libraries, custom rRNA probes were designed by the manufacturer using small and large ribosomal subunit sequences reconstructed with EMIRGE [19] from our 16 metagenomes. For this, EMIRGE was run over 40 iterations with clustering at 97% identity and using the SILVA 132 database [6]. Ribosomal sequences generated were then used as target sequences in the design of custom AnyDeplete probes using NuGEN's proprietary algorithm. 2×125 bp reads were generated using the Illumina HiSeq 2500 V4 platform. Adapter removal and quality trimming followed methods for metagenomic reads. Residual rRNA sequences were removed using SortMeRNA v2.1 [20]. Reads were then mapped to contigs from the dereplicated MAGs using Bowtie2 v2.3.5 [21] (--end-to-end --very_sensitive). Read counts were determined using featureCounts v1.6.3 [22] (-F SAF). Singleton reads per gene were removed. Read counts were normalized via the transcripts per kilobase per million reads mapped (TPM) formula with library size: (number of reads mapped to gene)*(1000/gene length)*(1000000/library size). EdgeR package v3.32.1 [23] was used to calculate log fold changes in gene expression between groundwater conditions based on unnormalised transcript counts.

Supplementary Results

Filter size fraction analysis

The large proportion of ultra-small microorganisms detected may be an underestimate, as we used 0.22 μm pore size filters, which can allow passage of some ultra-small cells [24], but facilitates quicker sample collection and hence greater biomass capture and faster RNA preservation than 0.1 μm filters. To account for potential loss, we compared the recovery of ultra-small phyla using filtration methods selecting for a wide range of size fractions (>1.5 nm, 0.1-0.22 μm, 0.2-1.2 μm and >1.2 μm) from un-enriched and particle-enriched groundwater (well E1). The highest recovery of ultra-small phyla, as a percentage of the total community (based on amplicons), was predictably when capturing all particles >1.5 nm using tangential flow filtration (Fig. S6). Relative abundances of ultra-small prokaryotes were 1.9-4.7 times lower in 0.1-0.22 μm, 0.2-1.2 μm and >1.2 μm groundwater fractions compared to bulk groundwater (> 1.5 nm). However, a substantial fraction was captured by combined 0.22 and 1.2 μm filters (there were only 1.0-1.3 times more ultra-small prokaryotes in the >1.5nm groundwater fraction than in the >0.22 μm fraction in terms of relative abundance). Moreover, ultra-small community composition in the previously collected samples (>0.22 μm) was more similar to bulk groundwaters than to other fractions (Fig. S6). Relative abundance values for all fractions were within the same range as those from our previously collected samples (>0.22 μm).

Newly-recovered ultra-small microbial genomes are phylogenetically unique

Based on relative genome abundance profiles, Patescibacteria were among the most abundant microbial phyla, on average, across sites (Fig. 1c), of which, Gracilibacteria was the fifth most abundant prokaryotic class, and genome nzgw366 (class ABY1) was the second most abundant genome overall. Our findings further illustrate the considerable diversity of Patescibacteria and other ultra-small prokaryotes in aquifer environments [25–27], and show that previously identified classes, families and genera are present in the studied aquifers (Fig. 3c). However, genomes here were all found to be unique species, as they shared <85% ANI with GTDB representative genomes (dereplicated at ≥99% ANI), much lower than the species level determination threshold of >96.5% ANI [28]. Furthermore, pairwise AAI values, between MAGs >80% complete and GTDB representatives, ranged from >87% to <40% (or 47% for best matches to GTDB genomes), and indicate that at least 73% of our ultra-small prokaryote genomes are novel genera (<65% AAI) (Fig. 3c)

[29]. Accordingly, GTDB-Tk based classification of all 216 genomes (using relative evolutionary divergence and ANI) could not resolve taxonomy for 44.7% at genus level (Fig. 3c), indicating a substantial fraction are novel groundwater genera.

Small genome sizes with limited metabolic capacities

MAGs ranged in estimated size from 0.32 Mbp (Patescibacteria nzgw479) to 9.69 Mbp (Planctomycetes nzgw518) (Fig. 3a). Overall, genomes of ultra-small prokaryotes were expectedly small (1 ± 0.4 Mbp on average) [30], within the range typically seen only in obligate symbionts [31]. In addition, we observed a wide range of genome G+C contents (24-62%). G+C proportions at the upper end of the range may be considered incompatible with rapid evolution of these organisms driven by genome reduction [26]. However, studies on symbiotic bacteria suggest that genome streamlining is not always followed by reduced G+C content as is universally believed [32, 33].

Analysis of Patescibacteria, Dependentiae and DPANN genomes predicted major deficits in biosynthetic capacities, supporting the view that most of these organisms live as symbionts [26]. For example, several enzymes involved in glycolysis were identified, yet the pathway could not be fully resolved (Fig. S4). Genomes also lacked genes required for the production of most amino acids, nucleotides, and co-factors, consistent with phylogenetically related ultra-small taxa from aquifers in the USA and Germany [30, 34]. DPANN archaea altogether possessed more biosynthetic potential than ultra-small bacteria (consistent with Castelle et al. [34]). We found DPANN had 47% more genes and 44% larger estimated genome sizes, while their genomes were estimated to be 6% less complete on average, than Patescibacteria (not Dependentiae) (Fig. S2). In addition, analysis of COG metabolic categories revealed that the metabolic capacities harbored by DPANN organisms differ substantially from their bacterial counterparts (Fig. 3b) [34].

Supplementary References

1. Rice EW, Baird RB, Eaton AD. Standard methods for the examination of water and wastewater, 23rd Edition. Journal of Chemical Information and Modeling. 2017.
2. Hugerth LW, Wefer HA, Lundin S, Jakobsson HE, Lindberg M, Rodin S, et al. DegePrime, a program for degenerate primer design for broad-taxonomic-range PCR in microbial ecology studies. Appl Environ Microbiol 2014;80:5116–5123.

3. Walters W, Hyde ER, Berg-Lyons D, Ackermann G, Humphrey G, Parada A, et al. Improved bacterial 16S rRNA gene (V4 and V4-5) and fungal internal transcribed spacer marker gene primers for microbial community surveys. mSystems 2015;1:e0000915.
4. Edgar RC. Search and clustering orders of magnitude faster than BLAST. Bioinformatics. 2010;26:2460–2461.
5. Edgar RC. UPARSE: highly accurate OTU sequences from microbial amplicon reads. Nat Methods. 2013;10:996–998.
6. Quast C, Pruesse E, Yilmaz P, Gerken J, Schweer T, Yarza P, et al. The SILVA ribosomal RNA gene database project: improved data processing and web-based tools. Nucleic Acids Res. 2013;41:D590–D596.
7. Bolyen E, Rideout JR, Dillon MR, Bokulich NA, Abnet CC, Al-Ghalith GA, et al. Reproducible, interactive, scalable and extensible microbiome data science using QIIME 2. Nat Biotechnol. 2019;37:852–857.
8. McMurdie PJ & Holmes S. phyloseq: An R package for reproducible interactive analysis and graphics of microbiome census data. PLoS ONE. 2013;8:e61217.
9. Oksanen J, Blanchet FG, Kindt R, Legendre P, Minchin PR, O'Hara RB, et al. Vegan: community ecology package software. 2016. https://github.com/vegandevs/vegan
10. Martin M. Cutadapt removes adapter sequences from high-throughput sequencing reads. EMBnet J. 2011;17:10–12.
11. Bankevich A, Nurk S, Antipov D, Gurevich AA, Dvorkin M, Kulikov AS, et al. SPAdes: a new genome assembly algorithm and its applications to single-cell sequencing. J Comput Biol. 2012;19:455–477.
12. Langmead B, Trapnell C, Pop M, Salzberg SL. Ultrafast and memory-efficient alignment of short DNA sequences to the human genome. Genome Biol. 2009;10:R25.
13. Kang DD, Li F, Kirton E, Thomas A, Egan R, An H, et al. MetaBAT 2: an adaptive binning algorithm for robust and efficient genome reconstruction from metagenome assemblies. PeerJ. 2019;7:e7359.
14. Wu Y-W, Tang Y-H, Tringe SG, Simmons BA, Singer SW. MaxBin: an automated binning method to recover individual genomes from metagenomes using an expectation-maximization algorithm. Microbiome. 2014;2:26.
15. Alneberg J, Bjarnason BS, de Bruijn I, Schirmer M, Quick J, Ijaz UZ, et al. Binning metagenomic contigs by coverage and composition. Nat Methods. 2014;11:1144–1146.
16. Sieber CMK, Probst AJ, Sharrar A, Thomas BC, Hess M, Tringe SG, et al. Recovery of genomes from metagenomes via a dereplication, aggregation and scoring strategy. Nat Microbiol. 2018;3:836–843.
17. Olm MR, Brown CT, Brooks B, Banfield JF. dRep: a tool for fast and accurate genomic comparisons that enables improved genome recovery from metagenomes through de-replication. ISME J. 2017;11:2864–2868.
18. Laczny CC, Sternal T, Plugaru V, Gawron P, Atashpendar A, Margossian HH, et al. VizBin - an application for reference-independent visualization and human-

augmented binning of metagenomic data. Microbiome. 2015;3:1.
19. Miller CS, Baker BJ, Thomas BC, Singer SW, Banfield JF. EMIRGE: Reconstruction of full-length ribosomal genes from microbial community short read sequencing data. Genome Biol. 2011;12.
20. Kopylova E, Noé L, Touzet H. SortMeRNA: fast and accurate filtering of ribosomal RNAs in metatranscriptomic data. Bioinformatics. 2012;28:3211–3217.
21. Langmead B, Salzberg SL. Fast gapped-read alignment with Bowtie 2. Nat Methods. 2012;9:357–359.
22. Liao Y, Smyth GK, Shi W. featureCounts: an efficient general purpose program for assigning sequence reads to genomic features. Bioinformatics. 2014;30:923–930.
23. Robinson MD, McCarthy DJ, Smyth GK. edgeR: a Bioconductor package for differential expression analysis of digital gene expression data. Bioinformatics. 2010;26:139–140.
24. Luef B, Frischkorn KR, Wrighton KC, Holman H-YN, Birarda G, Thomas BC, et al. Diverse uncultivated ultra-small bacterial cells in groundwater. Nat Commun. 2015;6:6372.
25. He C, Keren R, Whittaker M, Farag IF, Doudna J, Cate JHD, et al. Genome-resolved metagenomics reveals site-specific diversity of episymbiotic CPR bacteria and DPANN archaea in groundwater ecosystems. Nat Microbiol. 2021;6:354–365.
26. Castelle CJ, Banfield JF. Major New Microbial Groups Expand Diversity and Alter our Understanding of the Tree of Life. Cell. 2018;172:1181–1197.
27. Brown CT, Hug LA, Thomas BC, Sharon I, Castelle CJ, Singh A, et al. Unusual biology across a group comprising more than 15% of domain Bacteria. Nature. 2015;523:208–211.
28. Varghese NJ, Mukherjee S, Ivanova N, Konstantinidis KT, Mavrommatis K, Kyrpides NC, et al. Microbial species delineation using whole genome sequences. Nucleic Acids Res. 2015;43:6761–6771.
29. Konstantinidis KT, Rosselló-Móra R, Amann R. Uncultivated microbes in need of their own taxonomy. ISME J. 2017;11:2399–2406.
30. Kantor RS, Wrighton KC, Handley KM, Sharon I, Hug LA, Castelle CJ, et al. Small genomes and sparse metabolisms of sediment-associated bacteria from four candidate phyla. mBio. 2013;4:e0070813.
31. Moran NA, Bennett GM. The tiniest tiny genomes. Annu Rev Microbiol. 2014;68:195–215.
32. McCutcheon JP, McDonald BR, Moran NA. Origin of an alternative genetic code in the extremely small and GC-rich genome of a bacterial symbiont. PLoS Genet. 2009;5:e1000565.
33. McCutcheon JP, McDonald BR, Moran NA. Convergent evolution of metabolic roles in bacterial co-symbionts of insects. Proc Natl Acad Sci USA. 2009;106:15394–15399.

34. Castelle CJ, Brown CT, Anantharaman K, Probst AJ, Huang RH, Banfield JF. Biosynthetic capacity, metabolic variety and unusual biology in the CPR and DPANN radiations. Nat Rev Microbiol. 2018;16:629–645.

Supplementary Figures

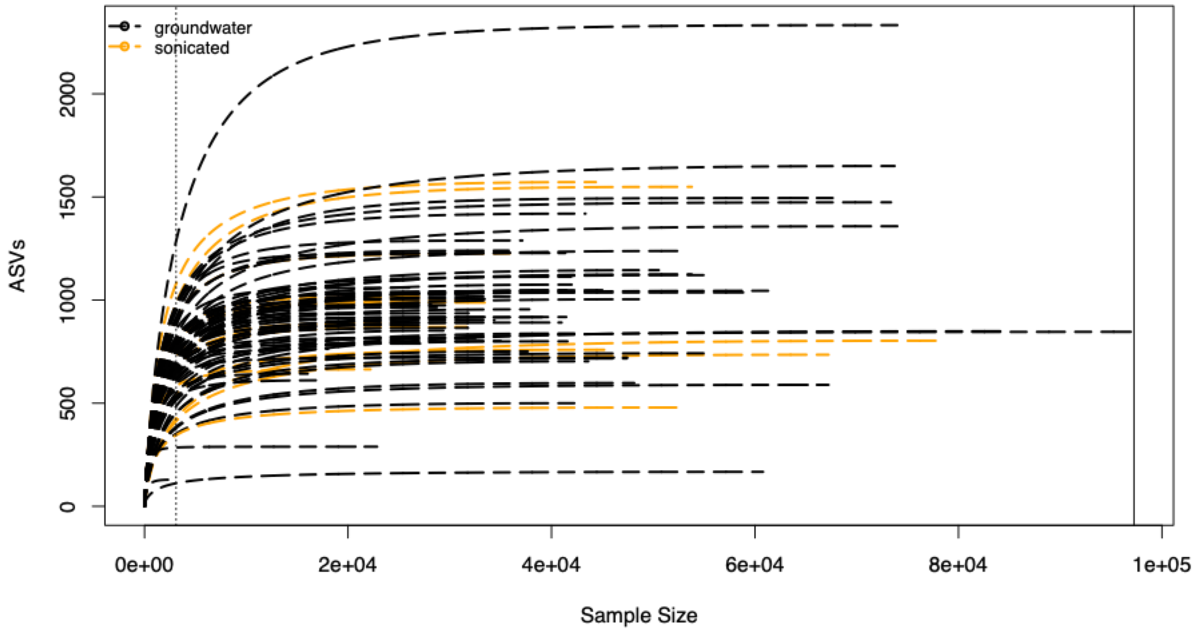

Figure S1. Rarefaction curves based on 16S rRNA gene ASVs. Curves show saturation of sampled variant diversity across all 81 groundwater samples and sediment-enriched groundwater.

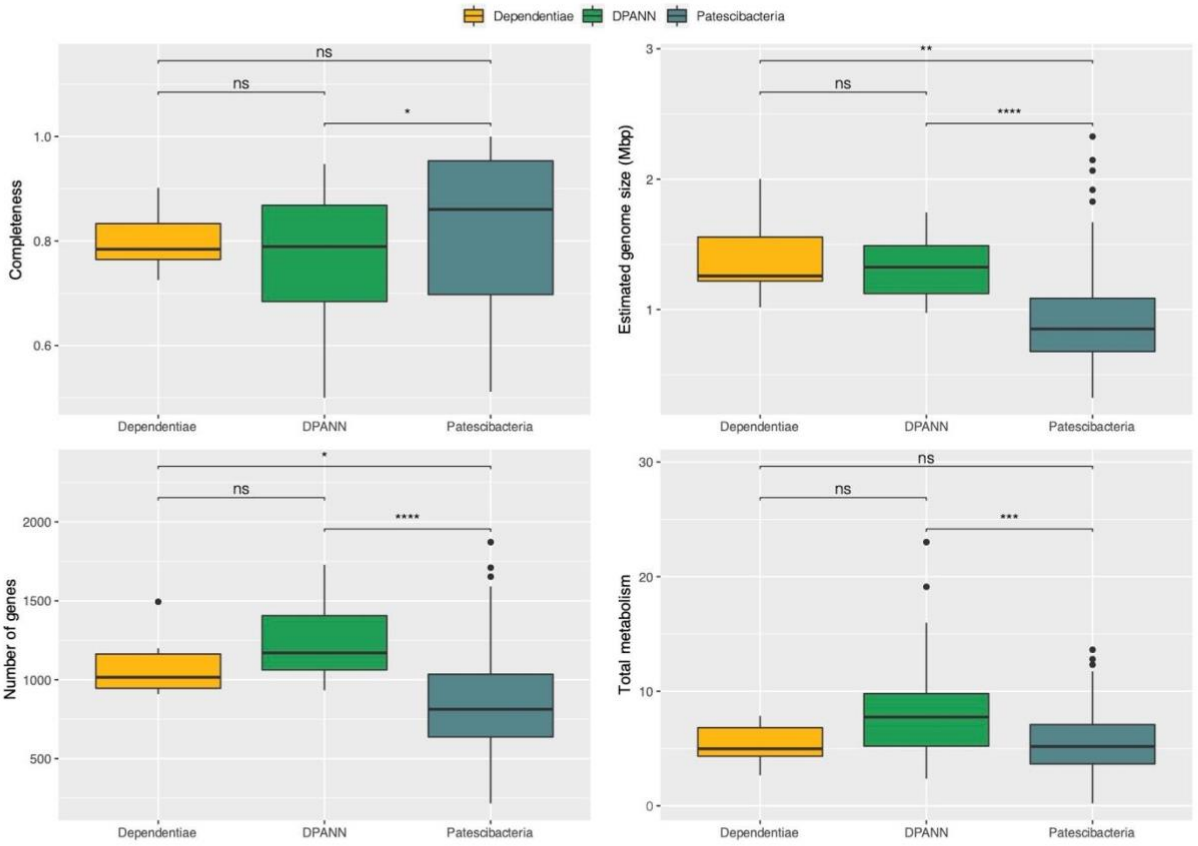

Figure S2. Genomic and metabolic attributes of the three ultra-small prokaryote groups identified in this study. Plots compare genome completeness (top left), estimated genome sizes (top right), number of predicted genes (bottom left), and total metabolic functions (bottom right). Total metabolism was calculated by adding the completeness values of each metabolic pathway presented in figure S3. Significance was tested using a Wilcoxon rank sum test (ns: $p>0.05$; *: $p<0.05$, **: $p<0.01$, ***: $p<0.001$; ****: $p<0.0001$).

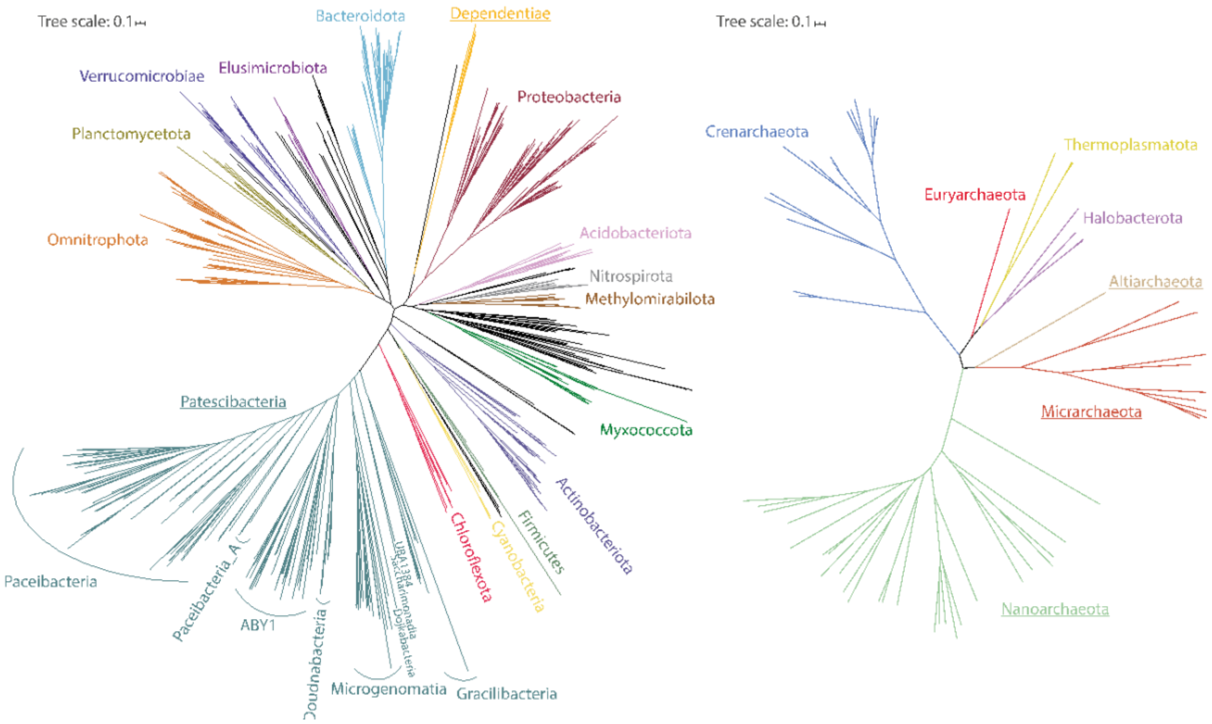

Figure S3. Unrooted maximum likelihood phylogenetic trees of 560 bacterial MAGs (left) and 66 archaeal MAGs (right) recovered in this study. Trees are based on either 120 concatenated bacterial marker genes or 122 concatenated archaeal marker genes from GTDB-Tk. Names of ultra-small microbial phyla are underlined. Scale bars indicate the number of substitutions per site.

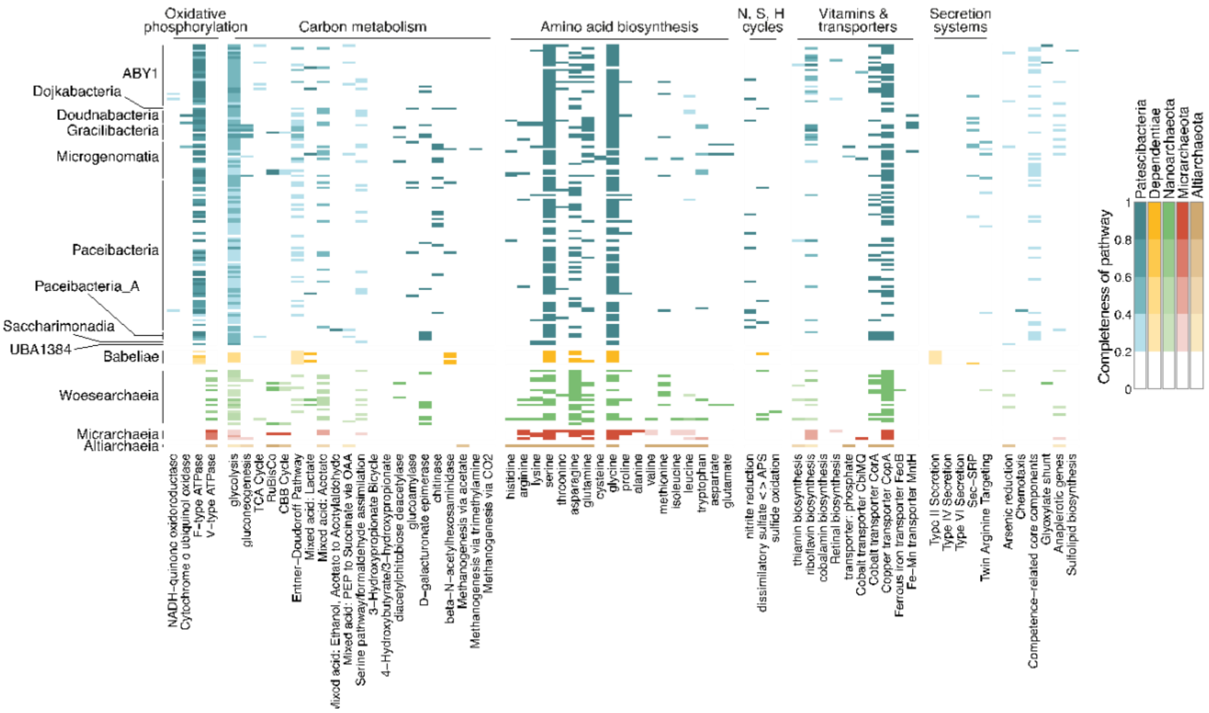

Figure S4. Heatmap showing the completeness of metabolic pathways (columns) encoded by the recovered Patescibacteria, Dependentiae and DPANN genomes (rows) (>70% completeness, <5% contamination). KEGG KO of genes required for various metabolic and biosynthetic functions shown here were parsed using KEGG-Decoder.

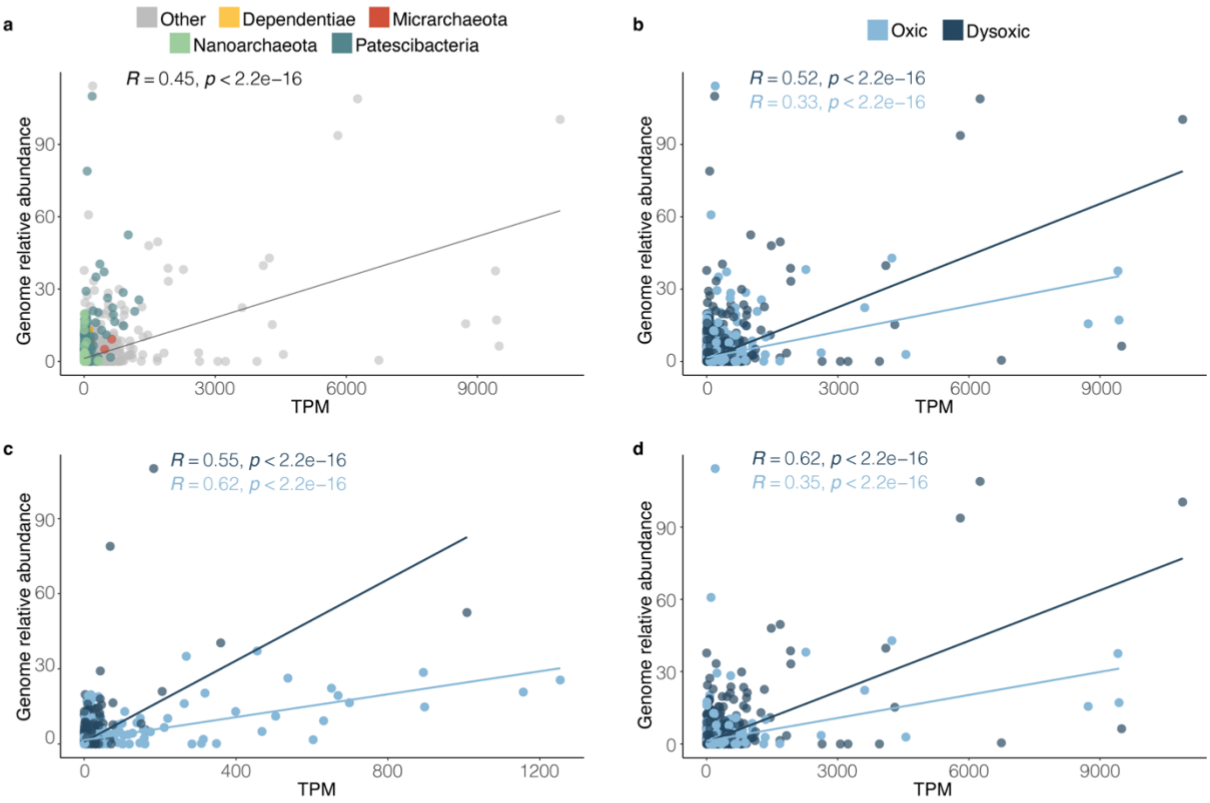

Figure S5. Correlations between genome relative abundance and total gene expression for each groundwater MAG across groundwater samples. Points are coloured by phylum (a), or by groundwater oxygenation regime (b) for all groundwater MAGs. Points are likewise coloured by oxygenation regime for ultra-small prokaryotic MAGs only (c), and other groundwater MAGs only (d). Correlation analyses were performed using Pearson correlation coefficient (R) and linear regression analysis using the ggscatter function from ggpubr R package.

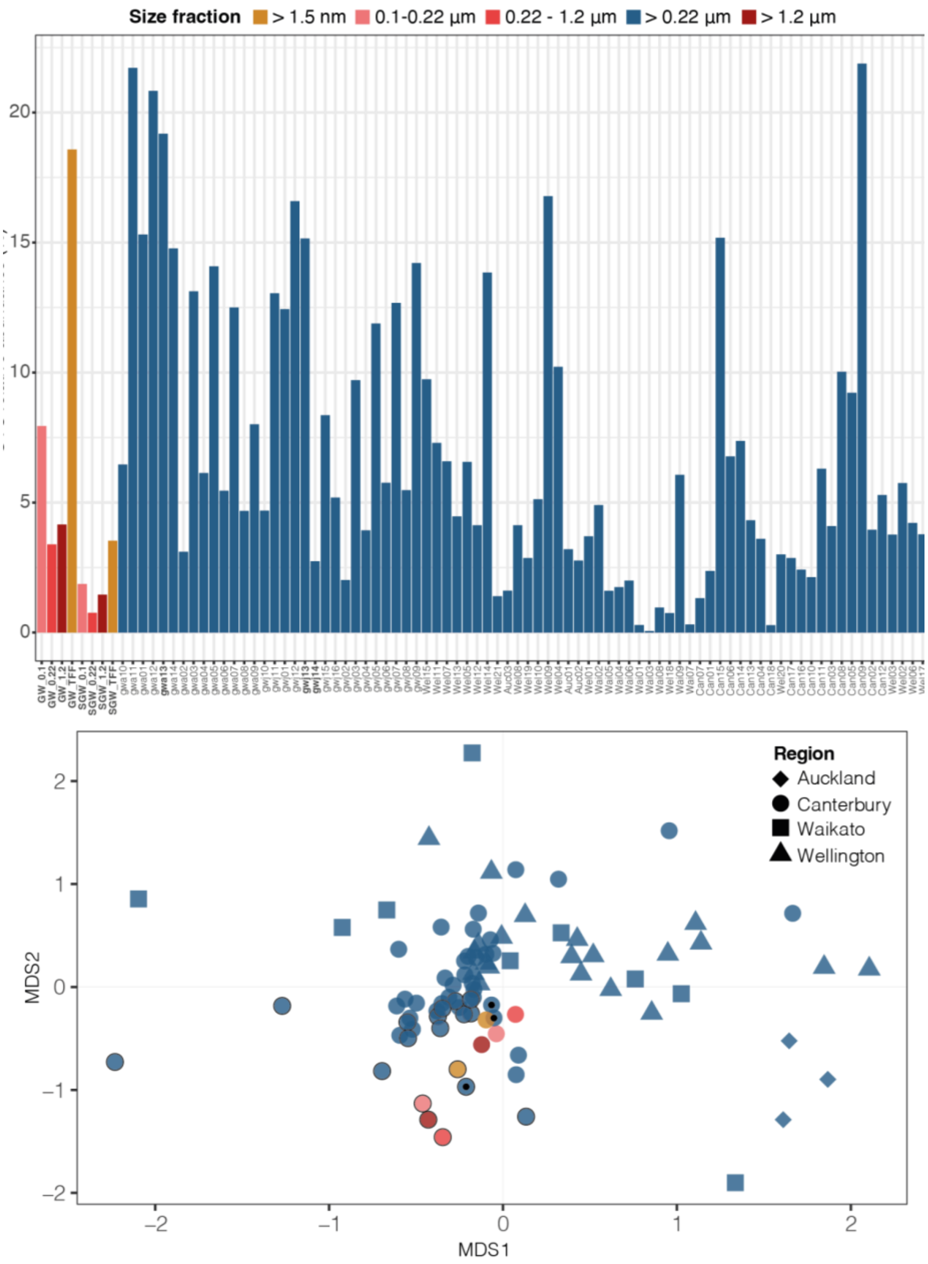

Figure S6. Ultra-small prokaryote relative abundance and composition based on groundwater filter size. (Top) Proportion of ultra-small prokaryote OTUs at each site based on 16S rRNA gene amplicons, according to the fraction size they were recovered from. Samples with name in bold were collected from the same well as size fraction series at different time points (Table S1). A filtration size of >1.5 nm was obtained by tangential flow filtration (TFF). Other filtration size ranges were obtained using flat mixed cellulose ester filters. GW = groundwater, and SGW = sediment (biomass) enriched groundwater (post sonication). (Bottom) Non-metric multidimensional scaling ordination of amplicon data based on Bray-Curtis dissimilarities. Analysis was performed using the vegan R package (metaMDS function, permutations = 999). Samples are colored based on groundwater size fraction. Symbols with darker centers correspond to samples collected from the same well as size fraction series at different time points. Points with darker rims correspond to sediment enriched samples.

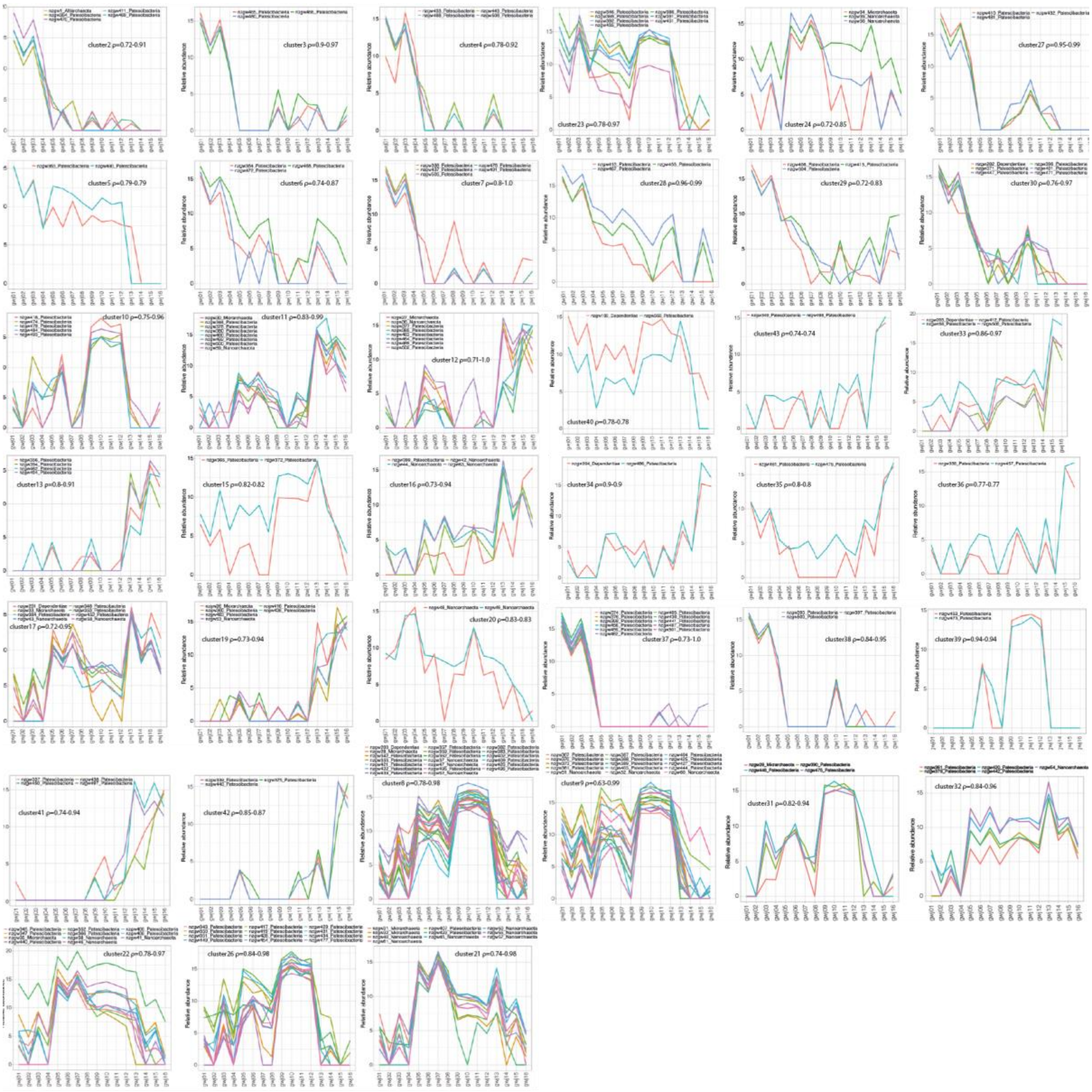

Figure S7. Genome relative abundance of members of 39 ultra-small microbial cohorts across 16 groundwater samples. Cohorts include 2 to 20 MAGs. Relative abundances are log normalized. Minimum and maximum Spearman's rank coefficients are given for each cohort or cluster.
